# Discovery of a Novel Tripeptide Linker that Broadens the Therapeutic Window of Auristatin-Based Antibody–Drug Conjugates by Reducing Bone Marrow Toxicity

**DOI:** 10.1021/acs.bioconjchem.6c00234

**Published:** 2026-07-23

**Authors:** Noah A. Bindman, Forgivemore Magunda, Vineet Kumar, Niels V. Rekers, David J. Ortiz, Esther S. Trueblood, Scott Blackburn, Roma Yumul, Divya Awasthi, Katie Caspary, Lauren Farr, Calvin Neace, Sarah Anderson, Brendan Drouhard, Xinqun Zhang, Hector Rincon-Arano, Matthew R. Levengood, Nicole M. Okeley, Peter D. Senter

**Affiliations:** † Pfizer, Inc. 21823 30th Dr SE, Bothell 98021, Washington, United States; ‡ Seagen, Inc. 21823 30th Dr SE, Bothell 98021, Washington, United States

## Abstract

Vedotin-based antibody–drug conjugates (ADCs) employing the valine–citrulline (Val–Cit) dipeptide linker and monomethyl auristatin E (MMAE) have achieved substantial clinical success but are frequently limited by dose-limiting hematologic toxicities, including neutropenia. These toxicities are hypothesized to arise, in part, from extracellular cleavage of the Val–Cit linker by neutrophil-derived proteases within the bone marrow microenvironment. Here, we describe the discovery and characterization of a tumor-selective tripeptide linker that improves the therapeutic index of MMAE-based ADCs. Using a high-throughput fluorescent peptide library screened against cancer and bone marrow homogenates, we identified peptide motifs preferentially cleaved in cancer lysates. Incorporation of these motifs into MMAE-containing drug linkers revealed a D-amino-acid–containing tripeptide (DLeu-Ala-Glu) that retained intracellular potency while exhibiting reduced susceptibility to neutrophil elastase and proteinase 3. ADCs bearing this linker demonstrated markedly reduced bone marrow toxicity in rats and cynomolgus monkeys relative to vedotin controls, with improved tolerability at higher doses and preserved antitumor efficacy across multiple xenograft models. Mechanistic studies showed that reduced linker proteolysis and increased linker hydrophilicity both contributed to reduced toxicity relative to vedotin. Together, these data demonstrate that rational peptide linker design integrating protease selectivity and physicochemical properties can substantially improve the safety profile of MMAE-based ADCs without compromising efficacy.

## Graphical abstract

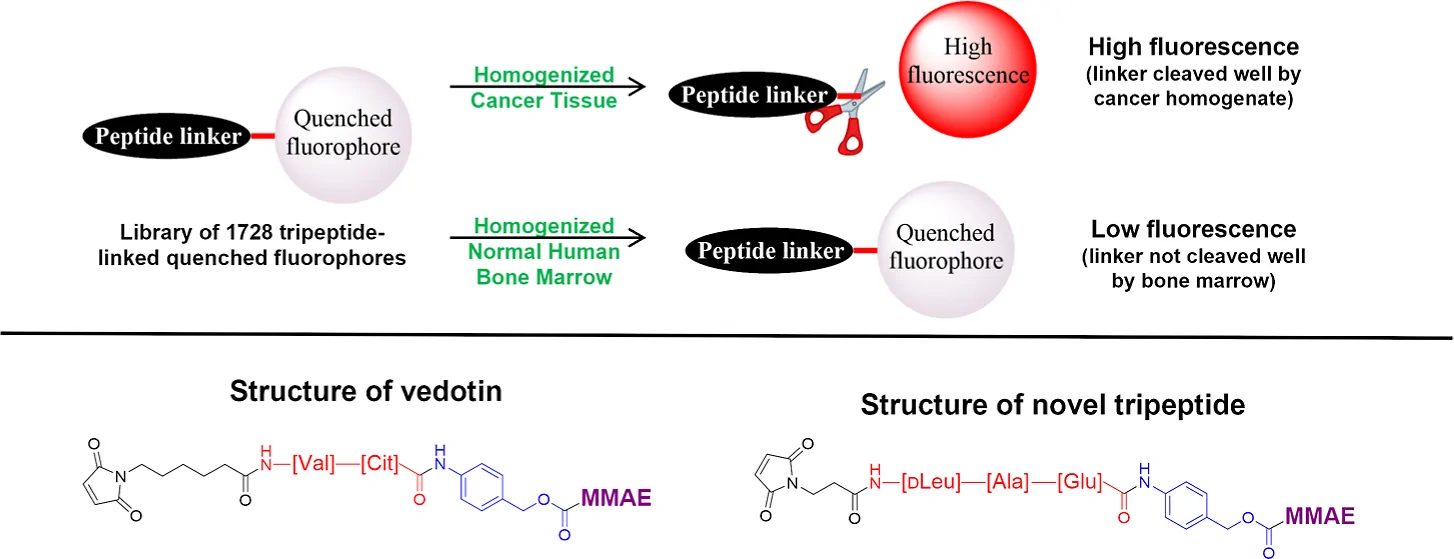

## Introduction

Antibody–drug conjugates (ADCs) employing the vedotin drug linker have demonstrated substantial clinical success,^1–4^ with 5 being FDA approved. Notably, the combination of enfortumab vedotin (Padcev) with pembrolizumab has received FDA approval for the treatment of advanced bladder cancer, nearly doubling median overall survival compared to standard chemotherapy.^5^ Despite these extraordinary outcomes, the dosage and duration of treatment with vedotin-based ADCs are constrained by off-target toxicities, including neutropenia and peripheral neuropathy.^6,7^ Strategies that reduce these toxicities while maintaining the effectiveness of vedotin ADCs would be a major advancement.

Vedotin ADCs utilize the cytotoxic agent monomethyl auristatin E (MMAE), conjugated to antibodies via a protease-cleavable valine–citrulline (Val–Cit) dipeptide linker, with an average drug-to-antibody ratio (DAR) of 4.^8,9^ Drug release primarily occurs following cellular internalization and lysosomal trafficking, where lysosomal proteases such as cathepsin B cleave the Val-Cit dipeptide, liberating MMAE, and resulting in direct tumor cell death, or indirectly through bystander activity.^10,11^ Nevertheless, a major limitation of the vedotin design is the insufficient specificity of the Val-Cit dipeptide linker for tumor cells, which can lead to drug release in normal tissues or plasma and subsequent toxicity. Furthermore, the bystander effect of MMAE amplifies this risk, as systemic drug release can cause widespread toxic effects. For instance, studies suggest that neutropenia associated with vedotin ADCs may result from extracellular cleavage of the Val-Cit linker by neutrophil proteases, or the fast clearance and intracellular processing of hydrophobic or high-DAR ADCs, leading to free MMAE release in the bone marrow and, due to MMAE’s bystander activity, causing subsequent hematopoietic progenitor toxicity and/or direct neutrophil toxicity.^12,13^

To overcome the toxicity limitations observed with current ADCs, novel cleavable peptide linkers are being developed to enhance ADC stability and specificity.^14–19^ These linkers aim to minimize premature cleavage in healthy tissues while maintaining efficient release in cancer cells. We hypothesized that substituting the Val-Cit dipeptide in vedotin with a tumor-selective peptide would reduce neutropenia and other toxicities without decreasing its therapeutic effectiveness. In this study, we describe the discovery of a new cleavable peptide linker that exhibits greater cleavage specificity in cancer cells than in bone marrow cells compared with the Val-Cit dipeptide. When incorporated into a full drug linker and conjugated to MMAE, this ADC exhibits significantly reduced bone marrow toxicity in preclinical models without compromising efficacy compared with a vedotin ADC.

## Results

### Synthesis of Fluorescent Peptide Library

To evaluate whether replacing the Val-Cit dipeptide with a novel cleavable peptide could mitigate the toxicity of vedotin ADCs without impacting efficacy, a fluorescent library comprising 1728 tripeptide linkers was synthesized using SPOT synthesis (Figure 1).^20,21^ Compared with commercial libraries at the time, which were limited to natural amino acids and lacking a *para*-aminobenzyl (PAB) group, this fluorescence assay-based library more accurately reflected the common structure of drug linkers, thereby enhancing the relevance of the results. Each linker incorporated a variable tripeptide, a self-immolative PAB spacer, and fluorescent molecule hydroxy coumarin. The tripeptides included hydrophobic, hydrophilic, charged, aromatic, and three nonproteinogenic amino acids. Additionally, during the synthesis, methionine, especially if in the P1 position, readily oxidized from the thioether to the sulfoxide. Proteolytic cleavage between the P1 position and the PAB group, followed by PAB self-immolation, released hydroxycoumarin, resulting in increased fluorescence emission at 450 nm.

**Figure 1:**
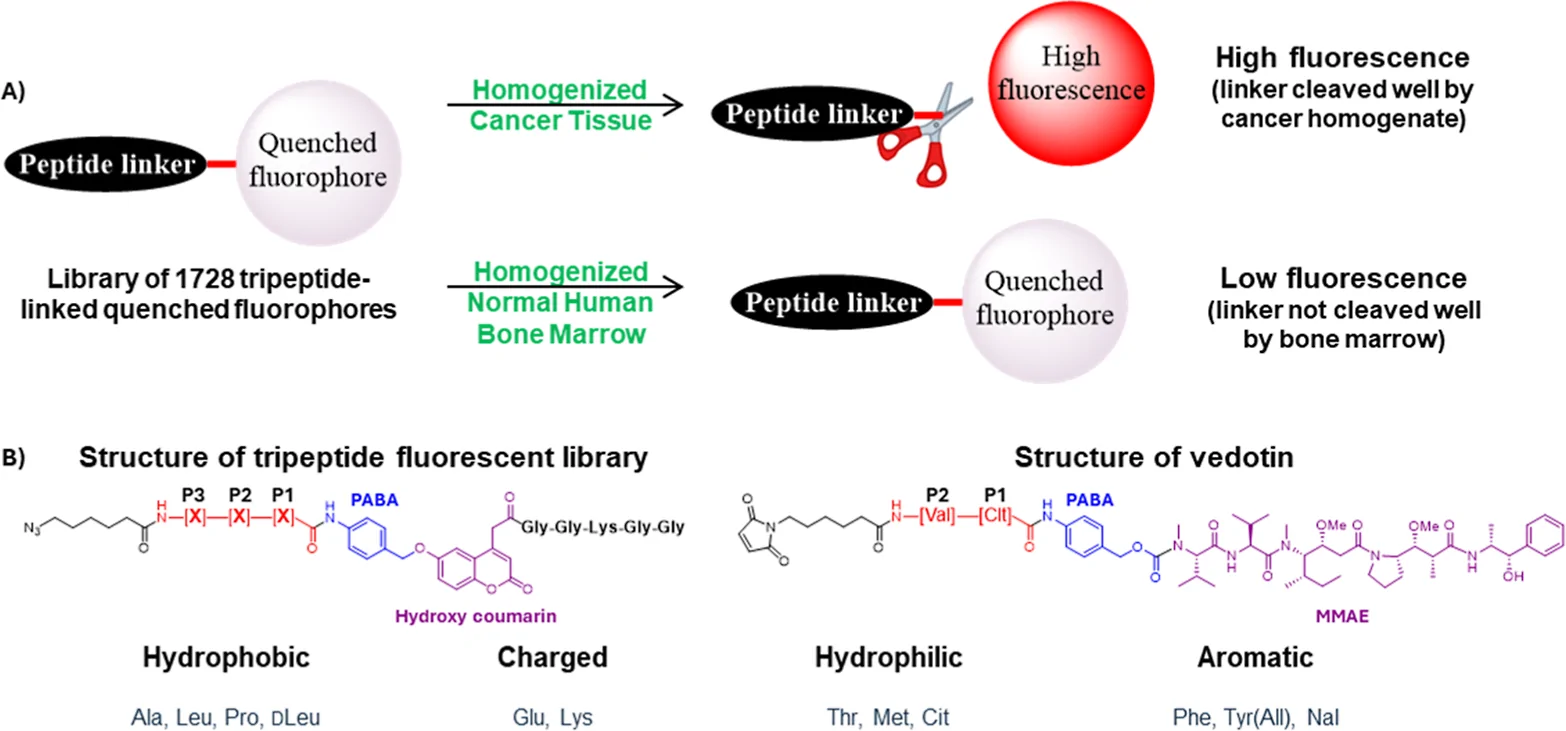
(A) Schematic of fluorescence assay designed to identify tripeptides preferentially cleaved in cancer cells over bone marrow cells. (B) Structure of fluorescence quenched tripeptide library compared to vedotin. Hydroxy coumarin becomes fluorescent upon proteolysis between P1 and PABA position. Unnatural amino acids are D-leucine (DLeu), citrulline (Cit), O-allyl-tyrosine (Tyr­(All)), and 2-naphthylalanine (Nal).

### High-Throughput Proteolysis Assay with Cancer and Bone Marrow Homogenates

We aimed to identify peptide sequences that were preferentially cleaved in cancer cells compared with bone marrow. To achieve this goal, homogenates from four cancer cell lines and human bone marrow were prepared in pH 4.5 buffer to mimic lysosomal conditions. Each tripeptide-fluorophore was incubated with both cancer and bone marrow homogenates for at least 4 h, after which tripeptide proteolysis was evaluated for selectivity for cancer over bone marrow homogenate. Among the top 100 sequences cleaved more efficiently in cancer homogenates (averaged across the four cell lines) versus bone marrow, tripeptides containing Glu, Cit, or Lys at the P1 position were significantly overrepresented (Figure 2A). Conversely, the P2 and P3 positions exhibit greater sequence variability. When analysis was restricted to tripeptides with low cleavage in bone marrow (defined as less than 80% of the average cleavage across all tripeptides), Met also appeared as an overrepresented residue at P1 (Figure 2b). On the basis of factors such as cancer selectivity, P1 diversity, and proteolysis rates, five lead tripeptides were chosen for further in vitro and in vivo testing. Additionally, one tripeptide, with minimal cleavage in both cancer and bone marrow homogenates, and vedotin were included for subsequent studies.

**Figure 2:**
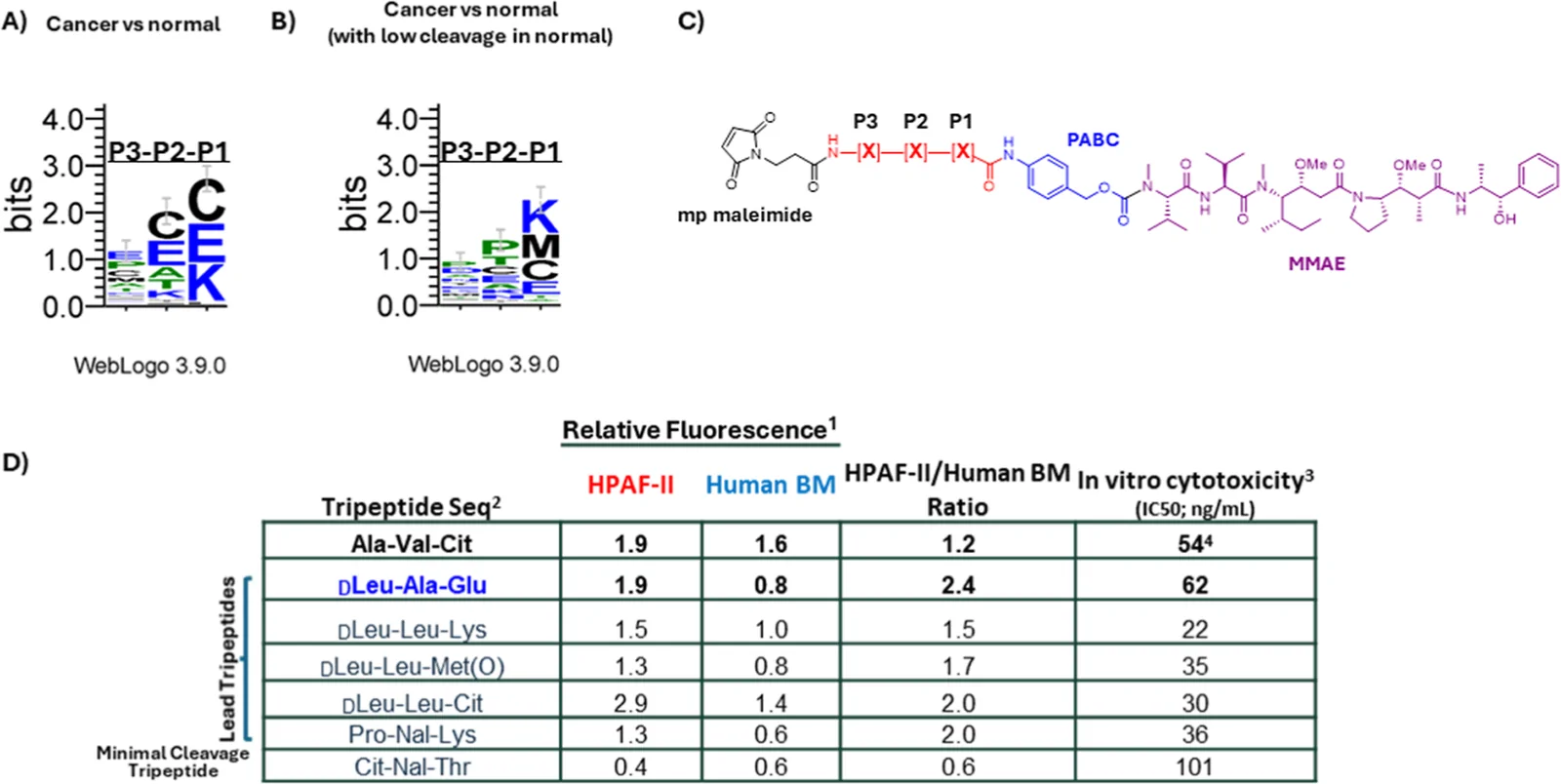
Frequency of amino acids in the P1, P2, and P3 positions of the top 100 tripeptides filtered by selective proteolysis in (A) cancer vs bone marrow or (B) cancer vs bone marrow when the intensity of proteolysis from bone marrow is ≤0.8-fold from the average cleaved tripeptide in bone marrow. (C) Structure of mp-tripeptide-MMAE drug linkers. (D) Fluorescence fold change and in vitro cytotoxicity of several tripeptide coumarin linkers or MMAE ADCs against the pancreatic adenocarcinoma cell line HPAF-II and human bone marrow. ^**1**^ Fluorescence fold change over average peptide in screen (1.0 = average fluorescence). ^**2**^ Natural amino acids are abbreviated with their traditional three letter codes (Cit = citrulline, Met­(O) = methionine sulfoxide, and Nal = 2-naphthylalanine. ^**3**^ Vedotin-like linkers were synthesized with the Val-Cit dipeptide substituted for a tripeptide and conjugated to an αvβ6-targeting hIgG1 antibody as a mixed 4-load. ^**4**^ Cytotoxicity of vedotin rather than Ala-Val-Cit-MMAE was measured against HPAFII cells.

### Cytotoxicity of Lead Tripeptide MMAE ADCs

To assess how novel tripeptides affect potency when compared to the Val-Cit dipeptide sequence, the tripeptide sequences were incorporated into MMAE-containing drug linkers using solid-phase peptide synthesis. These drug linkers contained maleimido propionic acid (mp) instead of the maleimido caproyl (mc) used in vedotin. This change was made to reduce maleimide deconjugation as the shorter length of the mp maleimide vs mc increases the rate of maleimide hydrolysis, stabilizing the linker to the antibody (Figure 2c).^22^ The linkers were conjugated as mixed 4-loads to an integrin β6-targeting antibody and tested for cytotoxicity against the αvβ6-expressing human pancreatic adenocarcinoma cell line HPAFII. The five lead ADCs, as described in Figure 2d, demonstrated cytotoxicity comparable to vedotin conjugated to the same antibody, whereas the tripeptide with low cancer proteolysis had ∼2-fold reduced cytotoxicity (Figure 2d), suggesting an association between the proteolysis assay and cytotoxicity.

### Rat Bone Marrow Toxicity of Tripeptide-MMAE ADCs

To assess if substituting the Val-Cit dipeptide with a novel tripeptide sequence can reduce the bone marrow toxicity observed with vedotin ADCs, the five lead tripeptide-MMAE drug linkers, the low cleavage tripeptide-MMAE, and vedotin were conjugated to a nontargeting hIgG1 antibody and administered to naïve female rats at a single IV dose of 10 mg/kg (Rat ETS1; Exploratory Toxicity Study 1 in Methods). The toxicity of these MMAE ADCs was also compared to hIgG1-mp-vcMMAE, a vedotin-like control ADC with an mp-maleimide to match the maleimide on the novel tripeptide ADCs, and to a vehicle control. Hematology and histology were evaluated 4 days postdose. As anticipated, rats treated with hIgG1-vedotin and hIgG1-mp-vcMMAE exhibited severe neutrophil and reticulocyte depletion (Figure 3a). Four of the six tripeptide ADCs showed comparable reductions in neutrophil and reticulocyte counts to hIgG1-vedotin. Conversely, hIgG1-DLeu-Ala-Glu and hIgG1-DLeu-Leu-Met­(O) had low incidence, minimal neutrophil reduction, and DLeu-Ala-Glu showed only minimal to mild depletion of reticulocyte counts compared to vehicle control by day 4. On histological analysis of the bone marrow core, as anticipated at this dose and schedule, hIgG1-vedotin showed a severe decrease in bone marrow cellularity characterized by loss of erythroid and myeloid hematopoietic cells and replacement with clear space and hemorrhage. Correlating with the hematology findings, hIgG1-DLeu-Ala-Glu and hIgG1-DLeu-Leu-Met­(O) showed minimal and mild decreased hematopoietic cellularity, confirming the reduced toxicity in these two groups compared to hIgG1-vedotin (Figure 3b).

**Figure 3:**
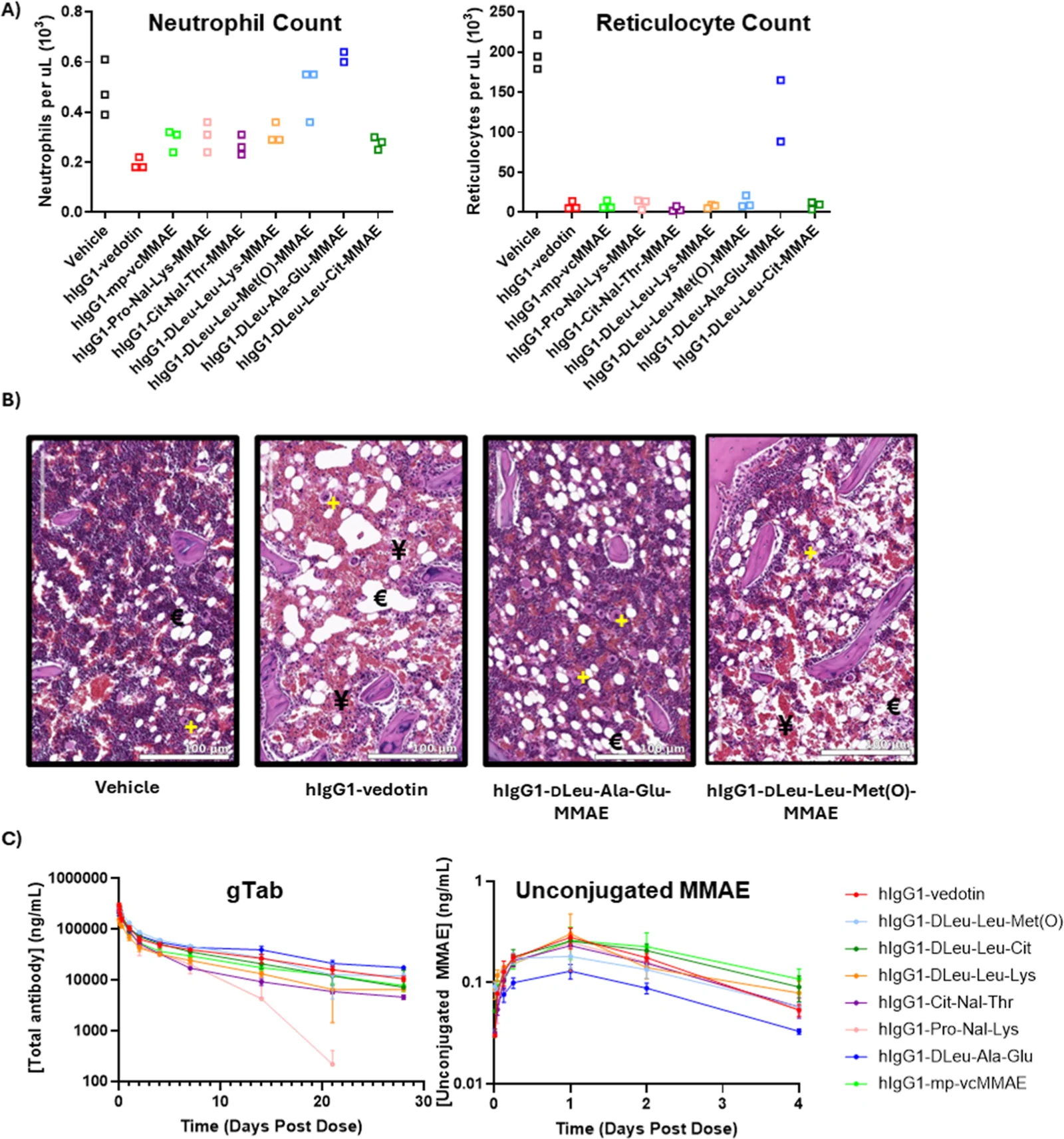
Comparison of hIgG1-tripeptide-MMAE ADCs to hIgG1-vedotin and hIgG1-mp-vcMMAE in a rat toxicity model at 10 mg/kg. *N* = 3 per group (A) Neutrophil and reticulocyte counts on day 4 post dose. (B) Histopathology of bone marrow on day 4. Small cells with dark blue nuclei and scant cytoplasm are myeloid and erythroid precursor hematopoietic cells. + = megakaryocytes, € = adipocytes. ¥ = hemorrhage and clear space. (C) Total antibody and unconjugated MMAE exposures of hIgG1-tripeptide-MMAE and Val-Cit-MMAE ADCs.

The pharmacokinetics (total antibody and unconjugated MMAE) of each ADC in the rat toxicity study were compared both among the tripeptide variants and against the two Val-Cit MMAE control ADCs (vedotin and mp-vcMMAE). Total antibody and unconjugated MMAE exposures were used to assess similarities in the nonspecific clearance, catabolism, and systemic drug concentrations. Most ADCs demonstrated comparable antibody pharmacokinetic profiles, with DLeu-Leu-Lys and Cit-Nal-Thr having approximately 2-fold reduced exposure (0–28d) (Figure 3c, Table S1). Notably, rats treated with the DLeu-Ala-Glu-MMAE ADC exhibited the lowest exposure of unconjugated MMAE (Figure 3c, Table S1), with a Cmax and AUC approximately 2-fold lower than those observed for hIgG1-vedotin.

Given the improved tolerability of hIgG1-DLeu-Ala-Glu-MMAE compared to hIgG1-vedotin, we dose escalated up to a maximum tolerated single dose of 40 mg/kg, an MTD 2.5-fold greater than vedotin ADC (rat ETS2). At this dose, hematologic evaluation indicated bone marrow toxicity characterized by decreased platelets, neutrophils, and reticulocytes on day 5 comparable to historical findings of hIgG1-vedotin dosed at its maximum tolerated dose of 15 mg/kg (Figure S1, Table S2).

### Non-Human Primate Toxicity of DLeu-Ala-Glu-MMAE ADC

Hematotoxicity is consistently observed as an antigen-independent dose-limiting toxicity across vedotin ADCs dosed in nonhuman primates.^23^ To establish whether the reduction in bone marrow toxicity observed in rat would translate to nonhuman primates, the toxicity of DLeu-Ala-Glu-MMAE and vedotin ADCs was assessed in cynomolgus monkey (NHP ETS1). Vedotin and DLeu-Ala-Glu-MMAE drug linkers were conjugated to a nontargeting hIgG1 antibody and were each administered to 3 male cynomolgus monkeys at 3 and 5 mg/kg and compared to a vehicle control group. As seen in rats, cynomolgus monkeys dosed with the DLeu-Ala-Glu-MMAE ADC showed reduced severity and incidence of bone marrow toxicity compared to those administered the vedotin ADC at a matched dose. At 7 days postadministration (study day 8), animals treated with 3 mg/kg vedotin ADC showed a 0.65x fold decrease in WBC with a 0.38x fold decrease in neutrophils compared to no change in the mean values in monkeys dosed with the matched doses of the DLeu-Ala-Glu-MMAE ADC relative to the baseline (day −5) mean value (Figure 4a). Additionally, animals treated with 5 mg/kg vedotin-ADC showed a statistically significant 0.59x fold decrease in WBC mean value relative to the baseline (day −5) mean value while the 0.65x change in monkeys dosed with matched doses of the DLeu-Ala-Glu-MMAE ADC was not statistically significantly different from its baseline (day −5) mean value. Animals treated with both test articles had comparable decreases in RBCs that were not statistically different from baseline mean values, and there were no changes in the reticulocytes compared to baseline (Figure 4a). The hematological changes in the vedotin ADC-treated monkeys correlated with minimal to marked decreased hematopoietic cellularity in the bone marrow with a shift toward less differentiated myeloid hematopoietic cells and generally decreased myeloid to erythroid ratio. Changes were noted in all 3 treated animals. Conversely, histopathological analysis revealed preserved to minimally reduced erythroid and myeloid lineages in the bone marrow of the DLeu-Ala-Glu-MMAE ADC-treated monkeys (Figure 4b). Pharmacokinetic profiling indicated comparable antibody-conjugated drug and unconjugated drug exposures between the two ADCs (Figure S2 and Table S3).

**Figure 4:**
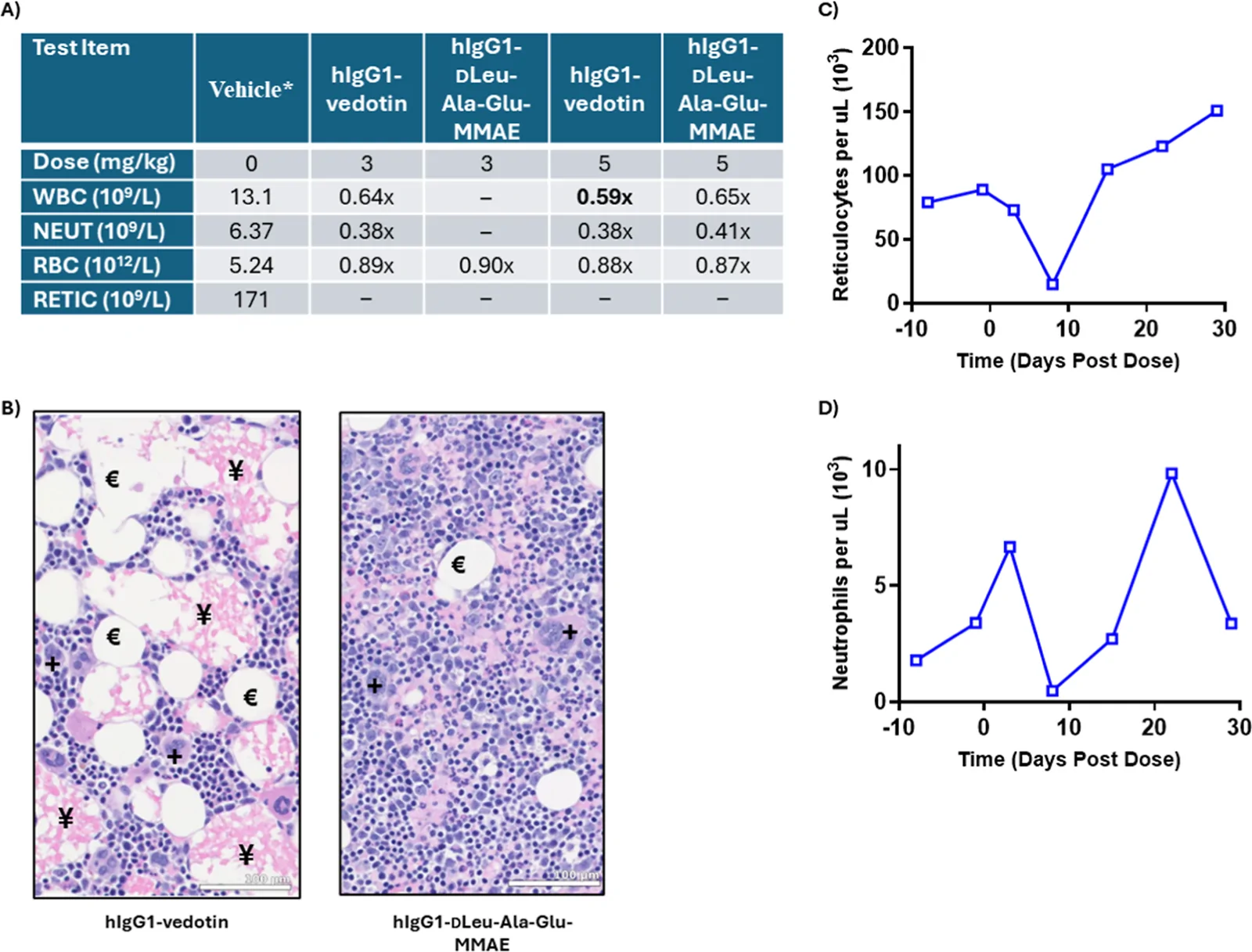
Toxicity comparison of hIgG1-vedotin and hIgG1-DLeu-Ala-Glu-MMAE ADCs dosed at 3 and 5 mg/kg in NHP (*N* = 3 males per group). (A) Test article-related hematology changes on day 7 post dose. A dash (−) indicates absence of change. Numerical values (x) indicate fold change of the treated group mean value relative to the baseline (day −5) mean value. * = Control group absolute mean values have been provided for reference. **Bolded** values indicate the mean value was statistically different from controls at *P* ≤ 0.05. (B) Histopathology of bone marrow at 5 mg/kg on day 7 showing reduced hematopoietic cellularity in hIgG1-vedotin. Small cells with dark blue nuclei and scant cytoplasm are myeloid and erythroid precursor hematopoietic cells. + = megakaryocytes, € = adipocytes. ¥ = hemorrhage and clear space. (C) Reticulocyte and (D) neutrophil counts after dosing NHP with 10 mg/kg ofan αLiv1-DLeu-Ala-Glu-MMAE ADC.

The DLeu-Ala-Glu-MMAE drug linker was then conjugated to an NHP-cross-reactive LIV-1 targeting antibody as a mixed 4-load and dosed in one cynomolgus monkey at 10 mg/kg (NHP ETS2) to evaluate the tolerability of the linker at doses above the MTD of a single dose of Liv-1-vedotin ADC (6 mg/kg). A 10 mg/kg dose of DLeu-Ala-Glu-MMAE ADC was tolerated. Hematological changes on day 7 included decreased red cell mass characterized by mildly decreased hematocrit (0.75–0.85x) and moderately decreased reticulocytes (0.1–0.2x) due to decreased erythropoiesis (Figure 4c). Neutrophils were transiently increased on day 3 and were moderately to markedly decreased by day 7 (Figure 4d). All changes recovered to the predose levels by day 15.

### Efficacy of Lead Tripeptide ADC Compared to Vedotin

To evaluate if the activity of the DLeu-Ala-Glu-MMAE drug linker was maintained compared to that of vedotin, cell-derived xenograft efficacy studies were conducted using ADCs targeting Trop2 and placental alkaline phosphatase (ALPP). Both the DLeu-Ala-Glu-MMAE and vedotin drug linkers were conjugated as mixed 4-loads to their respective antibodies and administered at matched doses and schedules across multiple human cell-line-derived xenograft models. In all models tested, the DLeu-Ala-Glu-MMAE ADCs demonstrated antitumor activity that was comparable to that of the vedotin-based ADCs (Figures S3 and S4), indicating that the improved safety profile of the DLeu-Ala-Glu linker did not compromise efficacy.

### Comparison of Cleavage of Tripeptide and Vedotin by Bone Marrow Proteases

To further investigate the mechanisms underlying the improved tolerability of the DLeu-Ala-Glu-MMAE drug linker vs vedotin, we assessed proteolysis of the respective linkers using proteases active in the bone marrow compartment. Neutrophil proteases such as neutrophil elastase are known to cleave the Val-Cit dipeptide of vedotin, leading to premature release of MMAE and likely contributing to neutropenia.^12^ To evaluate whether the DLeu-Ala-Glu-MMAE linker is similarly susceptible, vedotin and DLeu-Ala-Glu-MMAE payload-linkers were conjugated as 8-loads onto hIgG1 and the ADCs were incubated with the neutrophil proteases, neutrophil elastase, and proteinase 3, at pH 7.5 and 37 °C for 120 min. Mass spectrometry analysis revealed that Val-Cit was cleaved between valine and citrulline, releasing Cit-PABC-MMAE, while the DLeu-Ala-Glu tripeptide remained intact (Figure 5a,c). A control drug linker substituting the D-leucine with an L-leucine (LLeu-Ala-Glu-MMAE) was also synthesized and conjugated to hIgG1 as an 8-load. When this ADC with all L-amino acids was incubated with neutrophil elastase and proteinase 3, proteolysis between the Ala and Glu was observed, releasing Glu-PABC-MMAE and indicating that the D-amino acid in DLeu-Ala-Glu confers resistance to proteolysis (Figure 5c). The cancer cell cytotoxicity of Cit-PABC-MMAE, Glu-PABC-MMAE, and MMAE was compared and although MMAE was the most active, Cit- and Glu-PABC-MMAE were potent (Figure 5b). This result suggests that Cit- and Glu-PABC-MMAE are cell permeable and have bystander activity.

**Figure 5:**
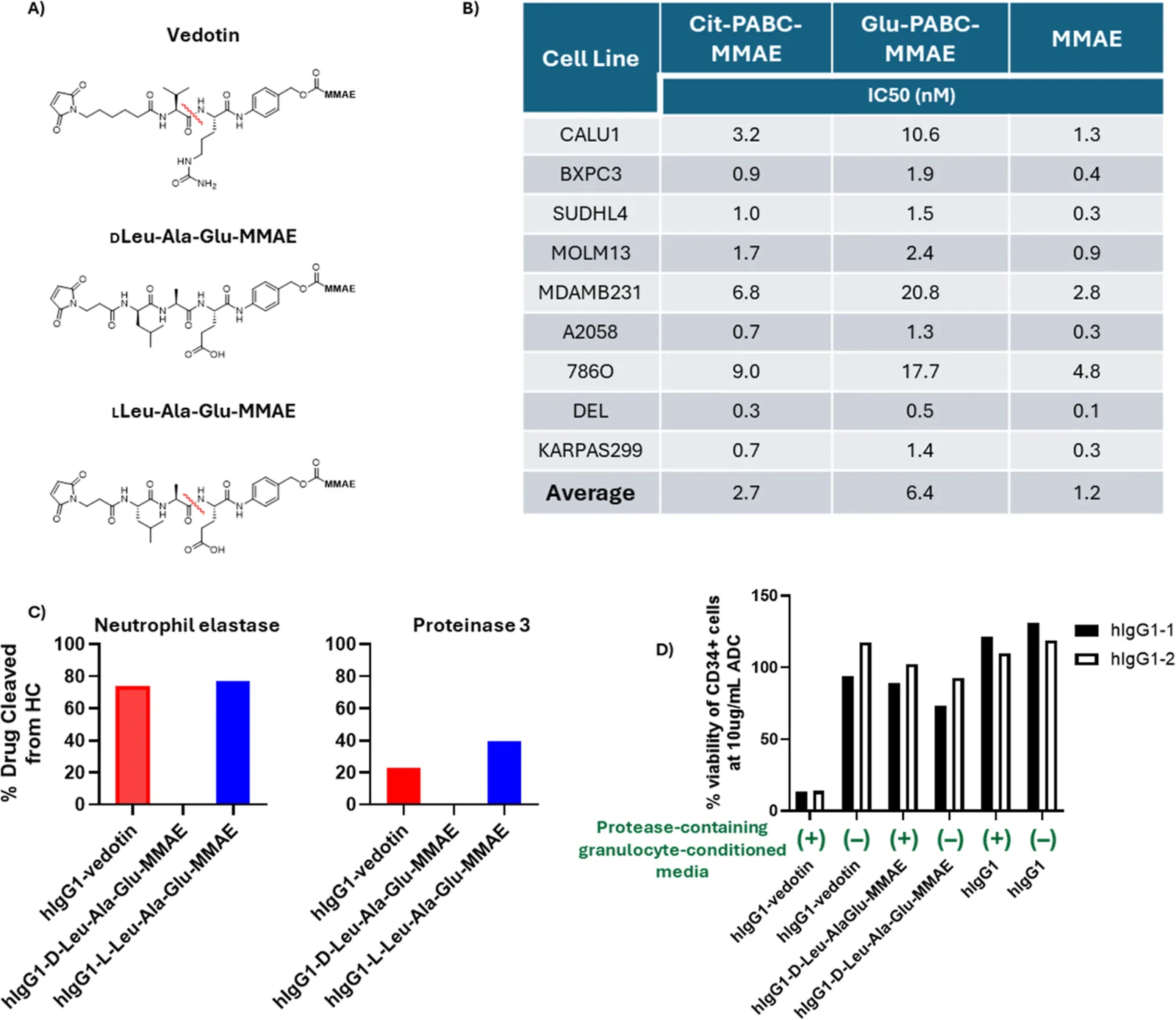
(A) Structures of MMAE drug linkers. Red line describes where proteolysis occurs after incubation by neutrophil elastase and proteinase 3. (B) Cytotoxicity (IC50) of Cit-PABC-MMAE, Glu-PABC-MMAE, and MMAE against various cancer cell lines and an average of all cell lines tested. (C) Proteolysis of vedotin, and tripeptide-MMAE ADCs by human neutrophil elastase and proteinase 3. Proteolysis of drug linker from the heavy chain was assessed. (D) Cytotoxicity of CD34+ cells after incubation of two hIgG1 DLeu-Ala-Glu-MMAE and vedotin ADCs with either granulocyte-conditioned media or blank media.

To further assess the effect of tripeptide linker proteolysis on bone marrow cells, vedotin and DLeu-Ala-Glu-MMAE linkers were each conjugated to two different nontargeting hIgG1 antibodies and were incubated with either neutrophil protease-containing granulocyte-conditioned media or blank media for 24 h at 37 °C. These mixtures were then applied to proliferating cultures of CD34+ hematopoietic cells to assess cytotoxicity. Vedotin ADCs incubated with granulocyte supernatant exhibited cytotoxicity, suggesting proteolytic cleavage of the Val-Cit dipeptide and drug release (Figure 5d). In contrast, DLeu-Ala-Glu ADCs incubated with granulocyte supernatant showed no cytotoxicity, indicating resistance to proteolysis. Neither vedotin nor DLeu-Ala-Glu-MMAE ADCs exhibited cytotoxicity on CD34+ cells after incubation in blank media.

### Nonspecific Binding and Acute Bone Marrow Toxicity

MMAE drug linkers with greater hydrophobicity are known to increase ADC clearance and nonspecific uptake in healthy tissue, including bone marrow, resulting in increased hematologic toxicity.^13,24^ The DLeu-Ala-Glu tripeptide introduces hydrophilicity to the drug linker through its negatively charged glutamic acid residue, potentially reducing ADC nonspecific binding relative to the more hydrophobic vedotin drug linker. To better understand the improved tolerability of the DLeu-Ala-Glu-MMAE ADC, we investigated the relationships between drug linker hydrophobicity, nonspecific binding, and bone marrow toxicity for tripeptide and vedotin ADCs. Nonspecific plasma binding was evaluated in vitro as a surrogate for nonspecific tissue binding. First, the DLeu-Ala-Glu-MMAE and vedotin drug linkers were conjugated as mixed 4-loads to a nontargeted hIgG1 antibody. The ADCs were fluorescently labeled then added to rat, cynomolgus monkey, and human plasma followed by incubation at 37 °C for 96 h (Figure 6a). Analysis by size exclusion chromatography with fluorescence detection revealed lower nonspecific binding for the DLeu-Ala-Glu-MMAE ADC compared with the vedotin ADC (Figure 6b). We next synthesized a panel of 34 tripeptide-MMAE drug linkers spanning a range of lipophilicities (calculated LogD @ pH 7.4; structures in Supporting Information), conjugated them to a nontargeted hIgG1 antibody as mixed 4-loads, and assessed nonspecific binding in rat plasma as before. A strong positive correlation was observed between drug linker hydrophobicity and nonspecific binding at 96 h with a Spearman correlation of *R* = 0.765 (Figure 6d). To determine whether this in vitro observation translated to bone marrow toxicity, each hIgG1-tripeptide-MMAE ADC was administered to rats as a single dose at 10 mg/kg and bone marrow toxicity was assessed via reticulocyte depletion at 96 h (Rat ETS3). Notably, ADCs with greater nonspecific binding exhibited greater reticulocyte depletion (Spearman correlation of *R* = 0.681), suggesting that linker hydrophobicity contributes to both nonspecific binding and hematologic toxicity (Figure 6e).

**Figure 6:**
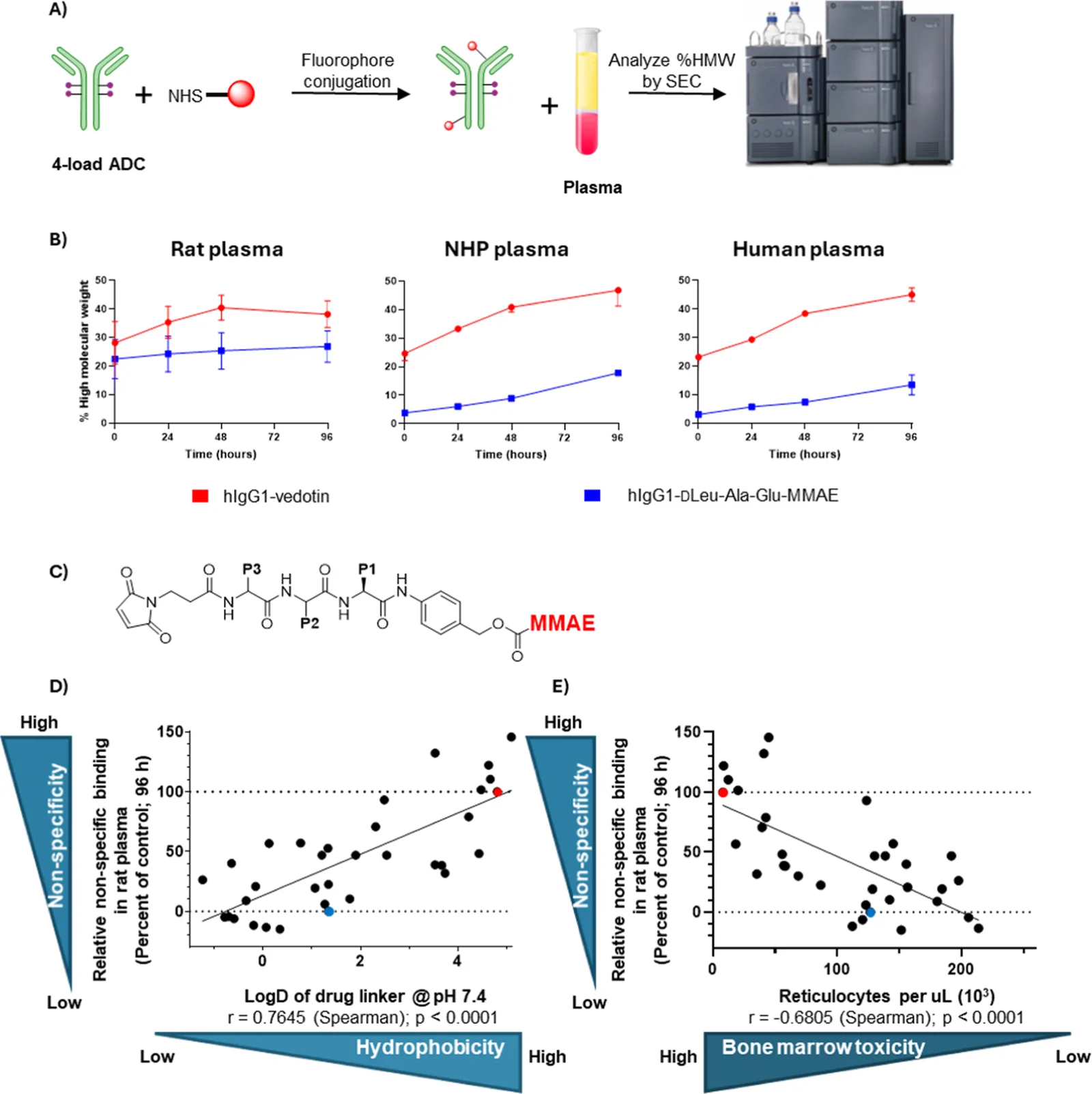
(A) Scheme depicting ADC nonspecific binding assay in plasma. (B) Nonspecific binding comparing a nontargeted DLeu-Ala-Glu ADC (blue) to a vedotin ADC (red) in rat, NHP, and human plasma. (C) Structure of tripeptide MMAE drug linkers used in graphs D and E. (D,E) Correlation between drug linker LogD @ pH 7.4 (calculated) and nonspecific binding (D) or bone marrow toxicity and nonspecific binding (E) of various tripeptide ADCs and vedotin. Vedotin (100% of control) is red circle, and DLeu-Ala-Glu-MMAE (0% of control) is blue circle. For rat toxicity, mean is graphed and *N* = 3 rats per group.

### Multidose Comparison of DLeu-Ala-Glu and LLeu-Ala-Glu

To disentangle the contributions of hydrophobicity and proteolytic susceptibility to bone marrow toxicity, we conducted a multidose rat study comparing hIgG1-DLeu-Ala-Glu-MMAE and hIgG1-LLeu-Ala-Glu-MMAE ADCs (q1wx4, 10 mg/kg; rat ETS4). These two linkers share similar hydrophobicity and nonspecific binding profiles (Figure S5) but differ in their susceptibility to neutrophil proteases (Figure 5c). ADCs generated by the two linkers had similar antibody exposure in rat (Figure 7a), but the DLeu-Ala-Glu-MMAE ADC had reduced unconjugated drug exposure (Figure 7b; Table S4), likely reflecting the relative resistance to serine protease cleavage. Following the first dose of the multidose rat toxicity study, hematology showed a modestly responsive increase in reticulocytes that did not significantly differ from the vehicle control in either linker (Figure 7c). However, following repeated weekly dosing, the LLeu-Ala-Glu ADC induced marked reductions in peripheral neutrophils and reticulocytes (days 22 and 28) that were more pronounced than in the hIgG1-DLeu-Ala-Glu-MMAE ADC. These hematological changes correlated with histopathological changes in the bone marrow characterized by decreased cellularity of myeloid and erythroid precursors that were minimal in rats administered the DLeu-Ala-Glu ADC and marked in those administered the protease-sensitive LLeu-Ala-Glu ADC (Figure 7d). These results, coupled with the data from Figure 6, suggest that while hydrophobicity may drive acute toxicity, proteolytic stability plays a critical role in mitigating delayed cumulative bone marrow injury.

**Figure 7:**
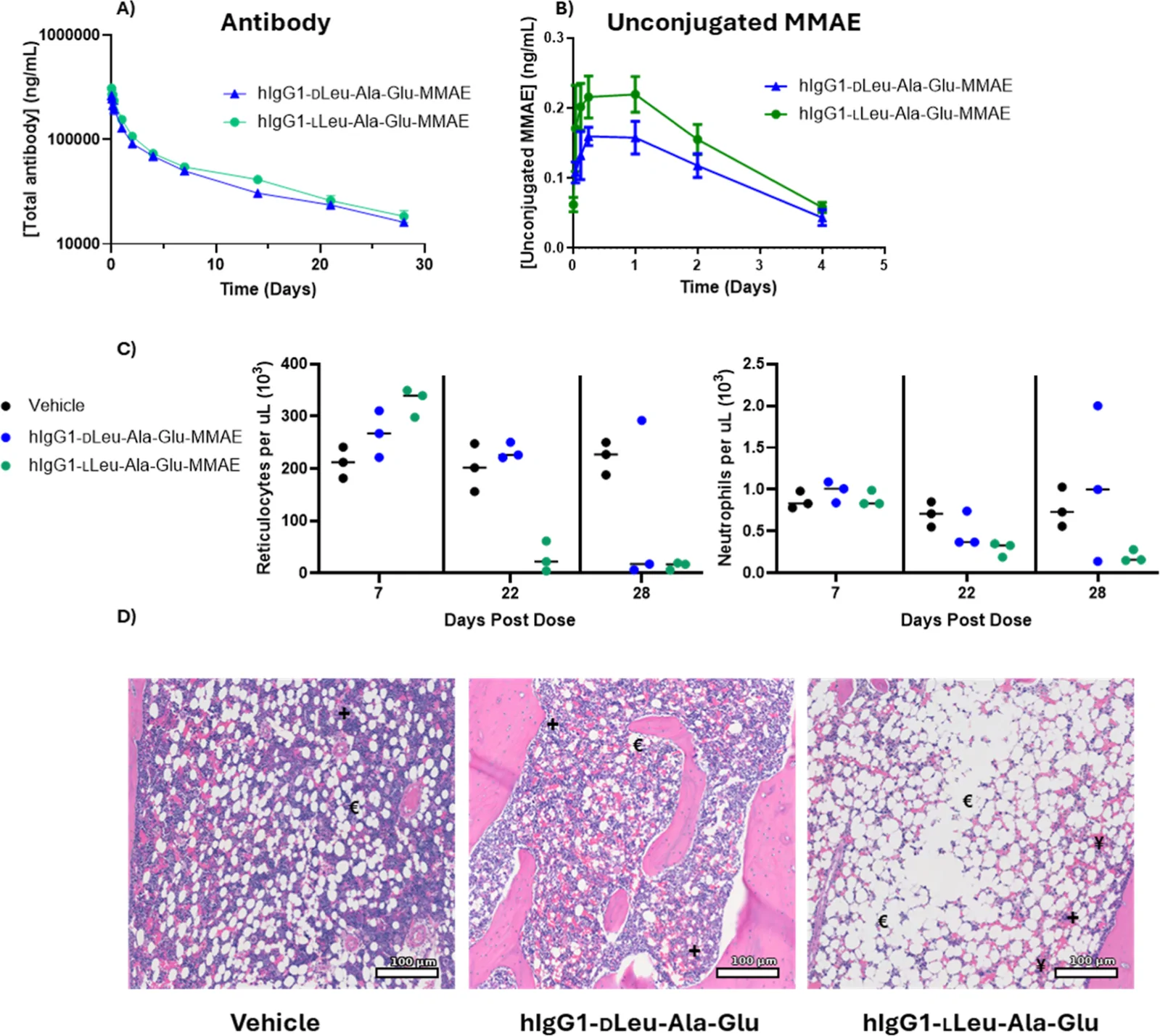
(A,B) Total antibody (A) and unconjugated MMAE (B) exposure after a single 10 mg/kg dose of ADCs in rat. Error bars represent standard error of the mean. (C) Reticulocyte (left) and neutrophil (right) concentrations on days 7, 22, and 28 postdose (10 mg/kg; QWx4). Line represents the median. (D) Histopathology of bone marrow 7-days post final dose (QWx4), showing reduced hematopoietic cellularity in hIgG1-LLeu-Ala-Glu compared to vehicle control and hIgG1-DLeu-Ala-Glu. *N* = 3 animals per group. Small cells with dark blue nuclei and scant cytoplasm are myeloid and erythroid precursor hematopoietic cells. + = megakaryocytes, € = Adipocytes. ¥ = hemorrhage and clear space.

## Discussion

The clinical utility of vedotin-based ADCs has been limited by dose-limiting neutropenia. A leading hypothesis suggests that this effect results from extracellular cleavage of the Val-Cit dipeptide linker by neutrophil-derived serine proteases, such as neutrophil elastase and proteinase 3. Under homeostatic conditions, the bone marrow is both the primary site of neutrophil production and a major site of their clearance, creating a local environment enriched in neutrophil-derived proteases.^25^ Supporting this hypothesis, a 2017 study by Zhao et al. demonstrated that vedotin ADCs, but not bystander-inactive MMAF conjugates, induced cytotoxicity in differentiating hematopoietic stem cells (HSCs), with unconjugated drug detected in the supernatant of neutrophil-conditioned media.^12^ These findings suggest that extracellular proteolysis of the Val-Cit linker within the bone-marrow microenvironment may contribute to the neutropenia observed in patients. Our study advances this mechanistic understanding by demonstrating that rational peptide linker design, guided by protease selectivity and hydrophilicity, can meaningfully improve the therapeutic index of MMAE-based ADCs.

A central theme emerging from our work is the critical role of protease selectivity in linker design. By leveraging a high-diversity peptide library, we identified tripeptide-based linker sequences that are efficiently cleaved in cancer lysates but more resistant to degradation by bone marrow proteases. Most notably, in vitro assays confirmed that the DLeu-Ala-Glu tripeptide confers resistance to neutrophil elastase and proteinase 3, in contrast to the canonical Val-Cit linker. The use of D-amino acids at the P3 position was particularly effective in disrupting recognition by neutrophil proteases, a finding that aligns with broader drug conjugate literature on the value of unnatural amino acids for proteolytic stability.^26–28^

More broadly, while a number of novel protease-cleavable peptide linkers have been described preclinically,^14–19^ clinical translation has remained limited. To the best of our knowledge, a legumain-cleavable peptide linker used in the ADC VIP943 represents one of the few examples to advance into clinical evaluation,^29^ while an ADC employing the Glu-Gly-Cit tripeptide (CBB-120) is in late preclinical work.^14^ These novel linkers highlight both the promise and challenges of expanding beyond Val-Cit. In contrast, clinically validated platforms such as deruxtecan-based ADCs employ the non-novel tetrapeptide Gly-Gly-Phe-Gly (GGFG) linker,^30,31^ which is efficiently cleaved by lysosomal proteases. Notably, emerging data suggest that the activity of GGFG-linked ADCs may, in part, arise from cathepsin L-mediated extracellular cleavage and subsequent bystander killing, underscoring the complex interplay between protease specificity, payload release, and permeability.^32^ These observations further emphasize the importance of designing linkers with defined proteolytic profiles to balance efficacy and systemic tolerability.

A major advance of this work is the demonstration that DLeu-Ala-Glu linkers broaden the therapeutic window of MMAE-based ADCs, being tolerated at doses exceeding those of vedotin in rodent and nonhuman primate models. Bone marrow hypocellularity/depletion was the predominant toxicological finding, with severity ranging from minimal to marked in a dose-responsive manner, while some additional tissue changes occurred under specific dosing conditions (Table S2). Crucially, these improvements in tolerability did not compromise the antitumor efficacy. Across multiple xenograft models, DLeu-Ala-Glu ADCs matched the activity of the vedotin-based controls.

Our data also reveal a strong correlation among linker hydrophilicity, reduced nonspecific binding, and bone marrow toxicity. ADCs incorporating hydrophilic linkers, such as DLeu-Ala-Glu, exhibited lower nonspecific binding in vitro and diminished bone-marrow toxicity across species. This relationship is mechanistically plausible as hydrophilic linkers may reduce steric repulsion and shield hydrophobic antibody patches, thereby minimizing off-target interactions. Importantly, our multidose studies attempted to disentangle the contributions of hydrophobicity and proteolytic susceptibility, showing that while hydrophobicity drives acute toxicity, proteolytic stability mitigates cumulative bone-marrow injury.

Integrating these findings, we propose a mechanistic model in which rational linker design, incorporating both protease selectivity and hydrophilicity, can decouple efficacy from toxicity in MMAE ADCs. The DLeu-Ala-Glu tripeptide emerges as a compelling alternative to Val-Cit, offering resistance to neutrophil protease cleavage, reducing systemic MMAE exposure, and improving bone marrow safety. These advances have direct clinical relevance as they address a major barrier to the broader utility of vedotin-based ADCs. Importantly, the tripeptide linker may not mitigate on-target, off-tumor toxicity compared to Val–Cit as this toxicity shares the same underlying mechanism as ADC efficacy, namely target-dependent internalization, linker proteolysis, and drug release.

Future studies should validate these findings in clinical settings and explore the generalizability of this design strategy across diverse payloads and targets. The mechanistic insights gained here may inform the next generation of ADCs, enabling safer and more effective therapies for patients with cancer.

## Methods

### General Information

All commercially available anhydrous solvents were used without further purification. The drug linker compounds were purified over a C12 Phenomenex Synergi 4 μm Max-RP 80 Å, LC column 250 mm of appropriate diameter eluting with 0.1% trifluoroacetic acid in water (solvent A) and 0.1% trifluoroacetic acid in acetonitrile (solvent B). The purification methods generally consisted of linear gradients of solvent A to solvent B, ramping from 90% aqueous solvent A to 10% solvent A. NMR spectral data were collected on a Varian Mercury 400 MHz spectrometer. Chemical shifts (δ) are given in ppm relative to TMS. Coupling constants (*J*) are reported in hertz.

### Tripeptide Library Synthesis and Cleavage

Peptide library members were synthesized on cellulose support using Fmoc SPOT synthesis with a key modification to facilitate downstream handling. Specifically, laser-perforated cellulose paper was used to define discrete circular synthesis zones, enabling the spatial separation of individual library members.

Following synthesis, each circular disc containing a unique peptide was manually punched out using a multichannel pipet and transferred into individual wells of a microtiter plate. The plate was then placed in a sealed ammonia chamber to cleave the synthesized tripeptides from the cellulose support. Cleaved peptides were solubilized in 50% aqueous acetonitrile and transferred to a fresh microtiter plate for a subsequent analysis.

#### Tissue Homogenization

Normal tissue or tumor tissue from mouse xenografts was suspended in buffer (50 mM Tris, 150 mM KCl, pH 7.0) and added to a tube containing Matrix D lysing beads (mpbio). The tissue was homogenized with a Precellys 24 homogenizer. The homogenized sample was centrifuged at 1000 *g* for 10 min, and the resulting supernatant was collected and then frozen at −80 °C until further use.

#### Fluorescence Assay

To a 384-well plate was added a mixture of tumor or bone marrow tissue homogenate and citrate buffer (100 mM, pH 4.5; 9 μL) followed by a fluorescently labeled library compound (1 μL; dissolved in 50% MeCN). The reaction was incubated at 37 °C, and fluorescence (330 nm excitation, 450 nm emission) was detected several times over a 4 to 6 h period. The fluorescence fold change was determined by dividing the fluorescence value at each time point with the background fluorescence when no homogenate was added.

#### Conjugation

The antibody in PBS with 2 mM EDTA, pH 7.4, was treated with 2.1 eq. TCEP (for 4-load ADCs) and then incubated at 37 °C for about 45 min. The MMAE drug linkers were then conjugated to the partially reduced antibody. Briefly, a 50% molar excess of drug-linker in DMSO was added to the reduced antibody in PBS/EDTA, with the final DMSO concentration adjusted to 10–20%. The reaction proceeded at room temperature for 30 min. To quench unreacted maleimide groups, an excess of QuadraSil MP was added. The resulting ADC was purified and buffer-exchanged into PBS using Sephadex G-25 desalting columns. The protein concentration was determined by absorbance at 280 nm, and the drug-to-antibody ratio (DAR) was quantified by HIC. Final ADC preparations were stored at −80 °C until use.

#### In Vitro Cytotoxicity

The cytotoxic effects of the ADCs were evaluated using a luminescent cell viability assay (CellTiter-Glo, Promega). Briefly, 40 μL of cell suspension containing approximately 400–1000 cells per well was seeded into 384-well opaque-walled plates. After cell attachment, 10 μL of either free drug or antibody–drug conjugate was added to the appropriate wells. Plates were incubated for 96 h at 37 °C in a humidified incubator with 5% CO_2_.

Following incubation, plates were equilibrated to room temperature for 30 min. An equal volume of the CellTiter-Glo reagent was then added to each well. The contents were mixed on an orbital shaker for 2 min to induce cell lysis, followed by a 10 min incubation at room temperature to stabilize the luminescent signal. Luminescence was recorded using a plate reader to quantify ATP levels as a surrogate for cell viability.

#### Proteolysis Assay of MMAE ADCs

A nontargeting hIgG1 antibody was fully reduced, and a drug linker was conjugated as an 8-load. A 20 μL of the mixture containing 500 ng/μL ADC and 10 ng/μL protease in 100 mM tris, 75 mM NaCl, pH 7.5 was prepared for each ADC. The reactions were incubated in 250 μL HPLC vials at 37 °C for 2 h and then immediately analyzed by a QToF mass spectrometer.

#### Proteolysis by CD34+ Granulocytes

Human bone marrow CD34+ cells from two different donors (Stemcell Technologies) were differentiated into myeloid progenitors using SFEM II media with myeloid expansion supplement (both from Stemcell Technologies) for 15 days. Cells were confirmed to be myeloid lineage by measurement of CD13 and CD15 by flow cytometry. Conditioned media from these donors was filtered through a 0.2 μm filter, and 20 μg/mL ADC or antibody was added to either conditioned media or untreated media for 24 h at 37 °C in a humidified incubator with 5% CO_2_. Cells from another human bone marrow CD34+ donor (Stemcell Technologies) were expanded using SFEM II with CD34+ expansion supplement (both from Stemcell Technologies) for 7 days. Cells were confirmed to have retained CD34+ expression by flow cytometry. These cells were plated at a density of 1000 cells/well in 384-well plates, and ADCs incubated in either conditioned media or untreated media were immediately added to the cells in a serial dilution. Plates were incubated for 96 h at 37 °C in a humidified incubator with 5% CO_2_. Following incubation, cell viability was measured as described above.

#### In Vitro Plasma Nonspecific Binding Assay

ADCs were labeled with Alexa Fluor 488 TFP ester (Molecular Probes), desalted, buffer exchanged into PBS, pH 7.4 (Gibco), and sterile filtered. The concentration and degree of labeling of the resulting ADC-AF488 conjugate was determined by UV absorbance prior to freezing at −80 °C. A degree of labeling of 2 to 3 was targeted to minimize in-assay artifacts. On the day of the experiment, AF488-ADC was diluted to 50 μg/mL in plasma and incubated at 37 °C. At the indicated time points, aliquots were analyzed in singlicate by SEC-UPLC with fluorescence detection. The resulting chromatograms were analyzed to determine % of high-molecular-weight species.

#### In Vivo Studies

All procedures performed on animals were in accordance with regulations and established guidelines and were reviewed and approved by an institutional animal care and use committee or through an ethical review process.

#### In Vivo Cytotoxicity

All in vivo experiments were approved by the Institutional Animal Care and Use Committee (IACUC) of Pfizer. Specific pathogen-free (SPF) mice were purchased from Inotiv (Indianapolis, IN, USA) or Jackson Laboratory (Sacramento, CA, USA) and housed in an SPF barrier facility. Tumor cell lines were tested for select pathogens and were found to be negative.

Female nude (Hsd/Athymic Nude-Foxn1nu, Inotiv) mice were implanted with 0.5 × 10^6^ FaDu (head and neck squamous cell carcinoma) or 5 × 10^6^ BxPC3 (pancreatic cancer) cells in 25% Matrigel HC subcutaneously. Once tumor volumes reached 100 mm^3^, mice were randomized into treatment groups and dosed with 1–3 mg/kg ADC every 7 days for three total doses. Tumor volumes were measured twice per week, and animals were euthanized when tumor volume reached 700–1000 mm^3^. Stock concentrations of ADCs were diluted to a desired concentration (with 0.01% Tween 20 in phosphate-buffered saline (PBS)) and injected intraperitoneally into each treatment group.

Female NOD/SCID/gamma KO (NSG, NOD.Cg-*Prkdc* ^scid^ *Il2rg* ^tm1Wjl^/Szj, Jackson Laboratory) mice were implanted with 5 × 10^6^ MCF7 (estrogen receptor-positive breast cancer) or 1 × 10^6^ MDA-MB-468 (triple negative breast cancer) cells in 25% Matrigel HC subcutaneously. Once tumor volumes reached 100 mm^3^, mice were randomized into treatment groups and dosed with either a single dose of 1–3 mg/kg ADC (MDA-MB-468 xenograft model) or 1–3 mg/kg ADC every 7 days for three total doses (MCF7 xenograft model). 24 h prior to receiving each ADC dose, each animal was treated with 10 mg/kg human intravenous immunoglobulin (hIVIG). Tumor volumes were measured twice per week, and animals were euthanized when tumor volume reached 700–1000 mm^3^. Stock concentrations of ADC were diluted to a desired concentration (with 0.01% Tween 20 in PBS) and injected intraperitoneally into each treatment group.

#### Analysis of In Vivo Antitumor Activity

Percent tumor growth inhibition (%TGI) was calculated on the day at which animals in the control group were terminated by the Jackson Laboratories method, defined as

$$\%TGI=100x(1-\left({meanvol}^{treated}/{meanvol}^{untreated}\right)$$

The analysis of the covariance (ANCOVA) test was used to determine whether a treatment elicited statistically significant antitumor activity compared with the untreated control group.

##### Toxicology Studies

The nonclinical safety assessment comprised four exploratory toxicology studies (ETS) conducted in Sprague–Dawley rats at Seagen, Inc. Two ETS were conducted in cynomolgus macaques at Charles River Laboratories (CRL) and Altasciences. All studies were designed to compare antigen-independent tolerability and bone marrow toxicity of antibody–drug conjugates (ADCs) with tripeptide linkers including DLeu-Ala-Glu–MMAE to vedotin-based ADCs with a valine–citrulline (Val-Cit)–linker. ADCs were conjugated to a nontargeting hIgG1 or αLiv1 antibodies. Test articles, dose, dose regimens, number of animals per study, and end points evaluated are shown in Table 1. Studies were conducted in compliance with applicable institutional animal care and use guidelines.

**Table 1:** Study Design Summary of Exploratory Toxicology Studies (ETS) in Rats and Cynomolgus Monkeys^a^

study ID	species (sex)	dosing regimen	test articles	dose level(s) (mg/kg)	group size (n)	end points
**Rat ETS1**	rat (female Sprague–Dawley)	single IV bolus (single dose)	vehicle hIgG1-vedotin hIgG1-mp-Val-Cit-MMAE) panel of 6 hIgG1-tripeptide-MMAE ADCs	0 (vehicle)10 mg/kg (all ADC groups)	6 females/group	hematology day 4 and necropsy day 28
**Rat ETS2**	rat (female Sprague–Dawley)	single IV bolus (single dose)	vehicle hIgG1-DLeu-Ala-Glu-MMAE among other test articles	vehicle 0 hIgG1-DLeu-Ala-Glu-MMAE40 mg/kg	1 female/group	hematology days 5, 8, 15, and 22 necropsy day 22
**Rat ETS3**	rat (female Sprague–Dawley	single-dose IV (in vivo)	panel of 34 tripeptide-MMAE linkers (mixed 4-loads on non-targeting hIgG1)	10 mg/kg (all ADC groups)	1 female/group	in vitro SEC-fluorescence monitored over 96 h; in vivo reticulocyte depletion assessed at 96 h postdose
**Rat ETS4**	rat (female Sprague–Dawley)	repeat-dose IV bolus Q1W × 4 (days 1, 8, 15, 22)	vehicle; hIgG1-vedotin.hIgG1-Leu-Ala-Glu-MMAE	10 mg/kg/dose (all ADC groups)	12 females/group; TK: 3 females (Group 4)	necropsy days 2, 8, 23, 29
**NHP ETS1 (CRL)**	cynomolgus monkey (male)	repeat-dose weekly IV bolus for up to 4 doses	vehicle hIgG1-vedotin. hIgG1-d-Leu-Ala-Glu-MMAE	vehicle-0; 3, 5 mg/kg (all ADC groups)	3 males/group	necropsy day 8, 15, 29
**NHP ETS2 (Altasciences)**	cynomolgus monkey (female)	two-phase DRF; single IV bolus on Day 1	includes hLIV22- DLeu-Ala-Glu-MMAE (phase I) among other test articles	hLIV22- DLeu-Ala-Glu-MMAE 10 mg/kg (single dose)	1 female	clinical pathology days 3, 8, 15, 22, 29, and 36)

a **Abbreviations:** ETS, exploratory toxicology study; IV, intravenous; Q1W × 4, once weekly for four doses; TK, toxicokinetics; acMMAE, antibody-conjugated MMAE.

Sprague–Dawley rats were obtained from approved commercial vendors and acclimated prior to study initiation. Animals were group-housed under controlled environmental conditions with ad libitum access to certified rodent diet and water, except where fasting was required for specific procedures. Cynomolgus macaques were socially housed where possible under controlled environmental conditions and provided a commercial primate diet with supplemental enrichment. All procedures were conducted in accordance with institutional animal care and use committee (IACUC) approvals at the respective CROs.

Animals were uniquely identified and randomly assigned to treatment groups and were observed at least once daily for mortality and clinical signs with additional observations performed around dosing. Body weights were recorded at scheduled intervals, including predose and terminal time points. In designated animals, blood samples were collected for clinical pathology evaluations, including hematology and serum chemistry, according to protocol-specified schedules.

Animals were euthanized at scheduled interim and terminal time points. Complete necropsies were performed, and selected organs including bone marrow core from the femur and sternum were collected, weighed where applicable, and preserved for microscopic evaluation. Histopathologic assessment was conducted on hematoxylin- and eosin-stained sections by a board-certified veterinary pathologist. Bone marrow cytology and evaluation of lymphoid and nonlymphoid tissues were included as specified in individual study designs. All study data were recorded, quality checked, and reported according to internal standard operating procedures. Descriptive statistics were used to summarize quantitative end points. Toxicologic interpretations were based on an integrated assessment of clinical observations, clinical pathology, toxicokinetics, and anatomic pathology findings across studies.

##### Safety/Hazard Statement

The study involved antibody–drug conjugates containing monomethyl auristatin E (MMAE), a highly potent cytotoxic payload. All handling of MMAE-containing materials was performed using appropriate containment and safety procedures to minimize exposure risk, in accordance with institutional guidelines for cytotoxic compounds.

No other significant or unusual hazards beyond standard laboratory practices were associated with the materials or procedures used. All work was conducted in accordance with institutional safety protocols using appropriate engineering controls and personal protective equipment (PPE) to minimize exposure including; formalin and xylene were used during tissue processing; both are toxic and require handling in a chemical fume hood with appropriate PPE (gloves, eye protection). Waste solvents were disposed of according to institutional hazardous waste protocols. All animal tissues were handled under BSL-2 conditions because of potential zoonotic risk. Procedures likely to generate aerosols were conducted in a certified biosafety cabinet.

## Supplementary Material



## Notes

Additional experimental details, including chemical syntheses and analytics (PDF)

## Abbreviations

ADC: antibody–drug conjugate
MMAE: monomethyl auristatin E
val-cit: valine-citrulline
FDA: U.S. Food and Drug Administration
DAR: drug to antibody ratio
PAB: *para*-aminobenzyl
P1, P2, P3: positions 1, 2, or 3 in the tripeptide sequence
PABC: *para*-aminobenzyl carbamate
mp: maleimidopropionyl
mc: maleimidocaproyl
hIgG1: human immunoglobulin G1
IV: intravenous
ETS: exploratory toxicology study
vcMMAE: val-cit-MMAE
Cmax: maximum concentration
AUC: area under the curve
MTD: maximum tolerated dose
mg/kg: milligram per kilogram
ng/mL: nanogram per milligram
WBC: white blood cell
RBC: red blood cell
NHP: nonhuman primate
Neut: neutrophil
retic: reticulocyte
Trop2: trophoblast cell-surface antigen 2
ALPP: placental alkaline phosphatase
ER+: estrogen receptor-positive
HNSCC: head and neck squamous cell carcinoma
TGI: tumor growth inhibition
ns: not significant
nM: nanomolar
LogD: logarithm of the distribution coefficient
NHS: *N*-hydroxysuccinimide
%HMW: percent high-molecular weight
μL: microliter
h: hour
q1wx4: dosing once per week, 4 times
MMAF: monomethyl auristatin F
HSC: hematopoietic stem cell
mm: millimeter
MeCN: acetonitrile
nm: nanometer
PBS: phosphate-buffered saline
EDTA: ethylenediaminetetraacetic acid
TCEP: tris­(2-carboxyethyl)­phosphine
DMSO: dimethyl sulfoxide
UV: ultraviolet
DRF: dose range finding
