# Supplementary material for: Discovery of a Novel Tripeptide Linker that Broadens the Therapeutic Window of Auristatin-Based Antibody–Drug Conjugates by Reducing Bone Marrow Toxicity
Source: Bioconjug Chem. 2026 Jul 23;37(8):1643–56. doi: 10.1021/acs.bioconjchem.6c00234 (PMC13495546; doi:10.1021/acs.bioconjchem.6c00234)
Supplement: Supplementary file 1 [file bc6c00234_si_001.pdf]

Supporting Information

Discovery of a novel tripeptide linker that broadens the therapeutic window of auristatin-based antibody-drug conjugates by reducing bone marrow toxicity

Noah A. Bindman[a†*], Forgivemore Magunda[a†], Vineet Kumar[a], Niels V. Rekers[a], David J. Ortiz[a], Esther S. Trueblood[b], Scott Blackburn[a], Roma Yumul[b], Divya Awasthi[b], Katie Caspary[a], Lauren Farr[a], Calvin Neace[a], Sarah Anderson[a], Brendan Drouhard[a], Xinqun Zhang[a], Hector Rincon-Arano[b], Matthew R. Levengood[a], Nicole M. Okeley[b], Peter D. Senter[a]

[a]Pfizer, Inc. 21823 30th Dr SE, Bothell, WA 98021, United States

[b]Seagen, Inc. 21823 30th Dr SE, Bothell, WA 98021, United States

[†]These authors contributed equally.

*Corresponding author. Email: noah.bindman@pfizer.com

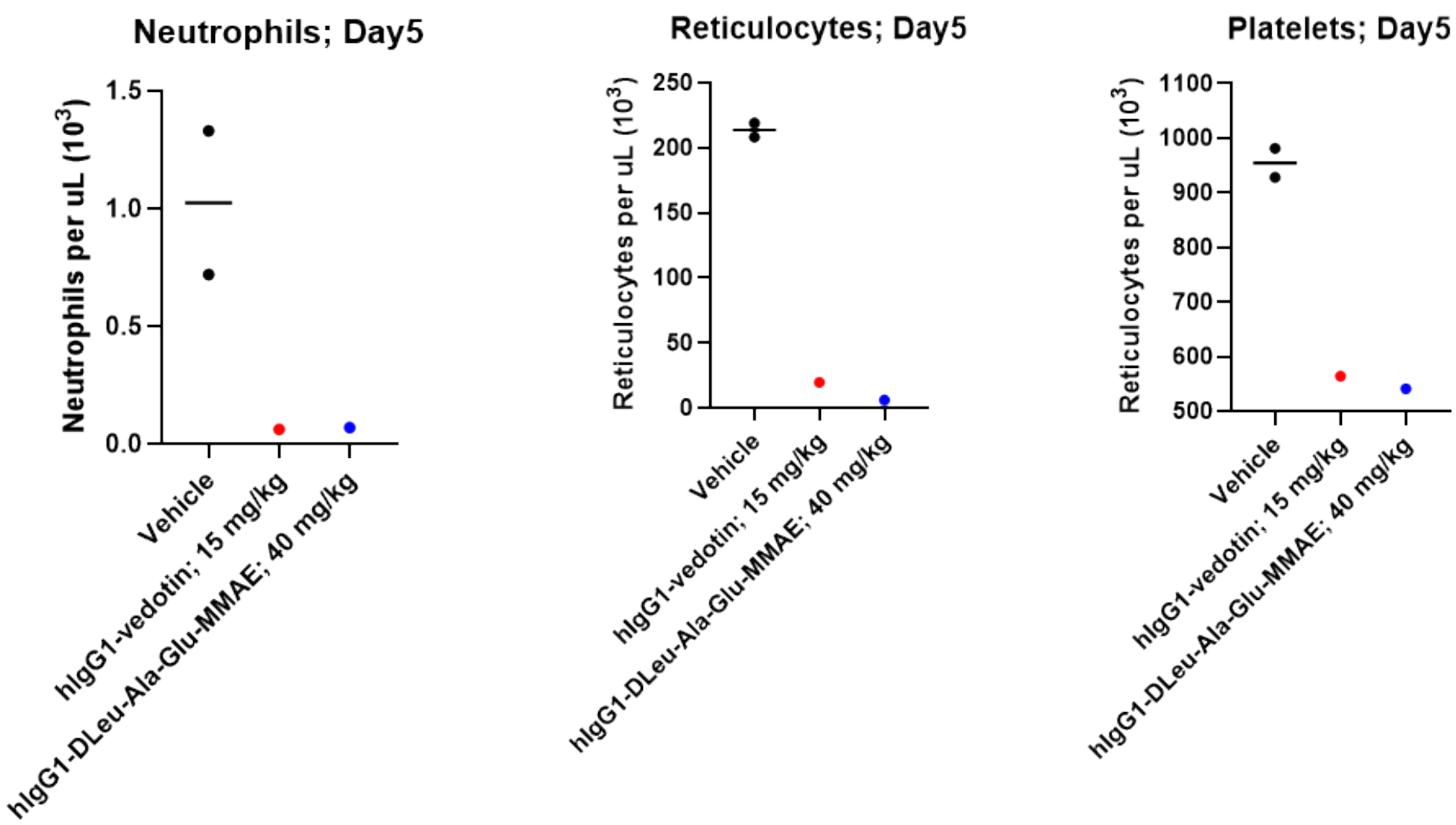

Figure S1. Neutrophil, reticulocyte, and platelet counts on day 5 comparing hIgG1-vedotin and hIgG1-DLeu-Ala-Glu-MMAE dosed at their MTDs of 15 mg/kg and 40 mg/kg, respectively. Line represents median. N = 1 animals per group for vedotin and DLeu-Ala-Glu-MMAE ADCs.

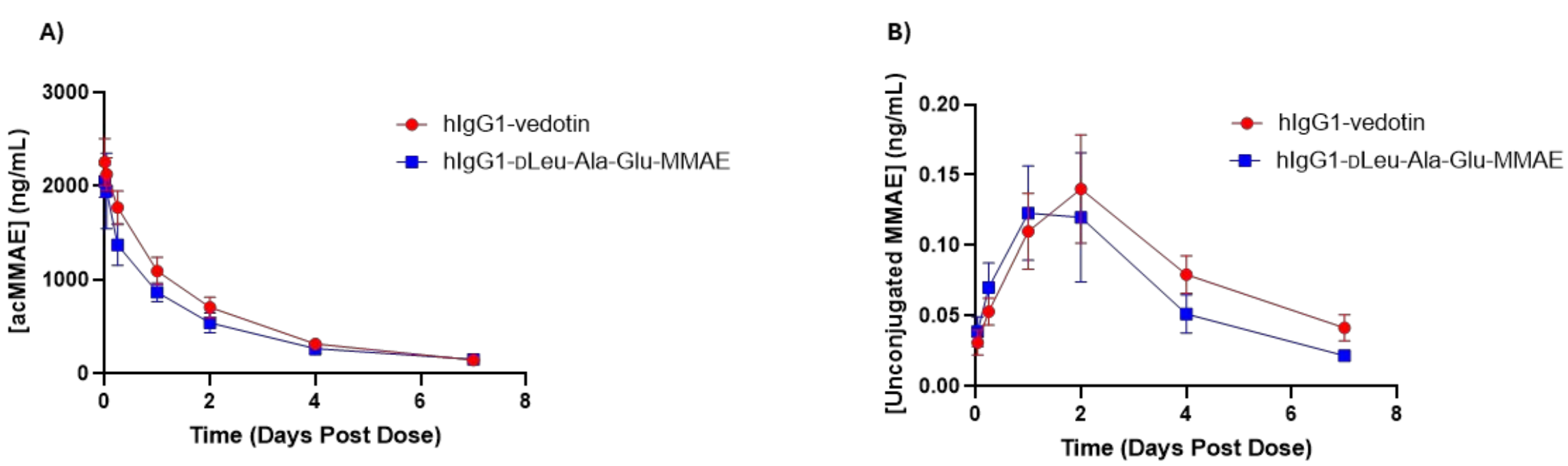

Figure S2. A) Antibody conjugated MMAE and B) unconjugated MMAE levels after non-human primates were dosed with 5 mg/kg of hIgG1-vedotin or hIgG1-DLeu-Ala-Glu-MMAE. Error bars represent standard error of the mean. N = 9 animals per group.

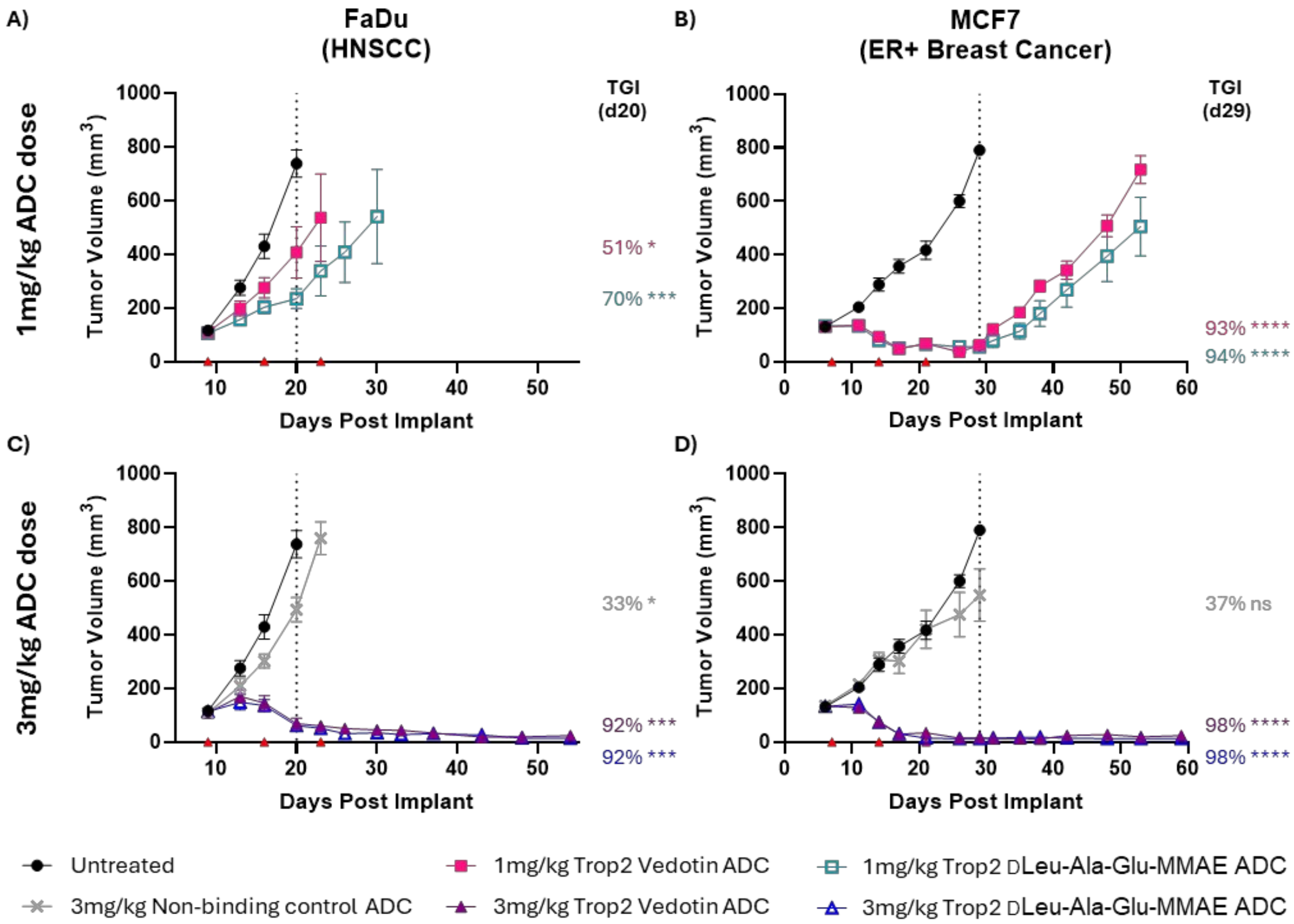

Figure S3. Comparison of the in vivo efficacy of vedotin and DLeu-Ala-Glu-MMAE ADCs in multiple cell-line derived xenograft models of HNSCC and ER+ breast cancer. (A,C) Mean tumor volume over time of FaDu tumor-bearing nude mice (n=5/group). (B,D) Mean tumor volume over time of MCF7 tumor-bearing NOD/SCID/gc KO (NSG) mice (n=5/group). All tumor-bearing mice were treated with 3 weekly doses of 1mg/kg (A,B) or 3mg/kg (C,D) of Trop2 vedotin, Trop2 DLeu-Ala-Glu-MMAE, or non-binding control ADC as indicated. All ADCs were mixed 4-load ADCs. Red arrows show timing of dose administration. Data is presented as mean ± SEM. Statistical analysis of antitumor activity was performed with ANCOVA test. ADC, antibody-drug conjugate; ER+, estrogen receptor-positive; HNSCC, head and neck squamous cell carcinoma; TGI, percentage tumor growth inhibition compared to untreated group; *, $p<0.05$; ***, $p<0.001$; ****, $p<0.0001$; ns, not significant.

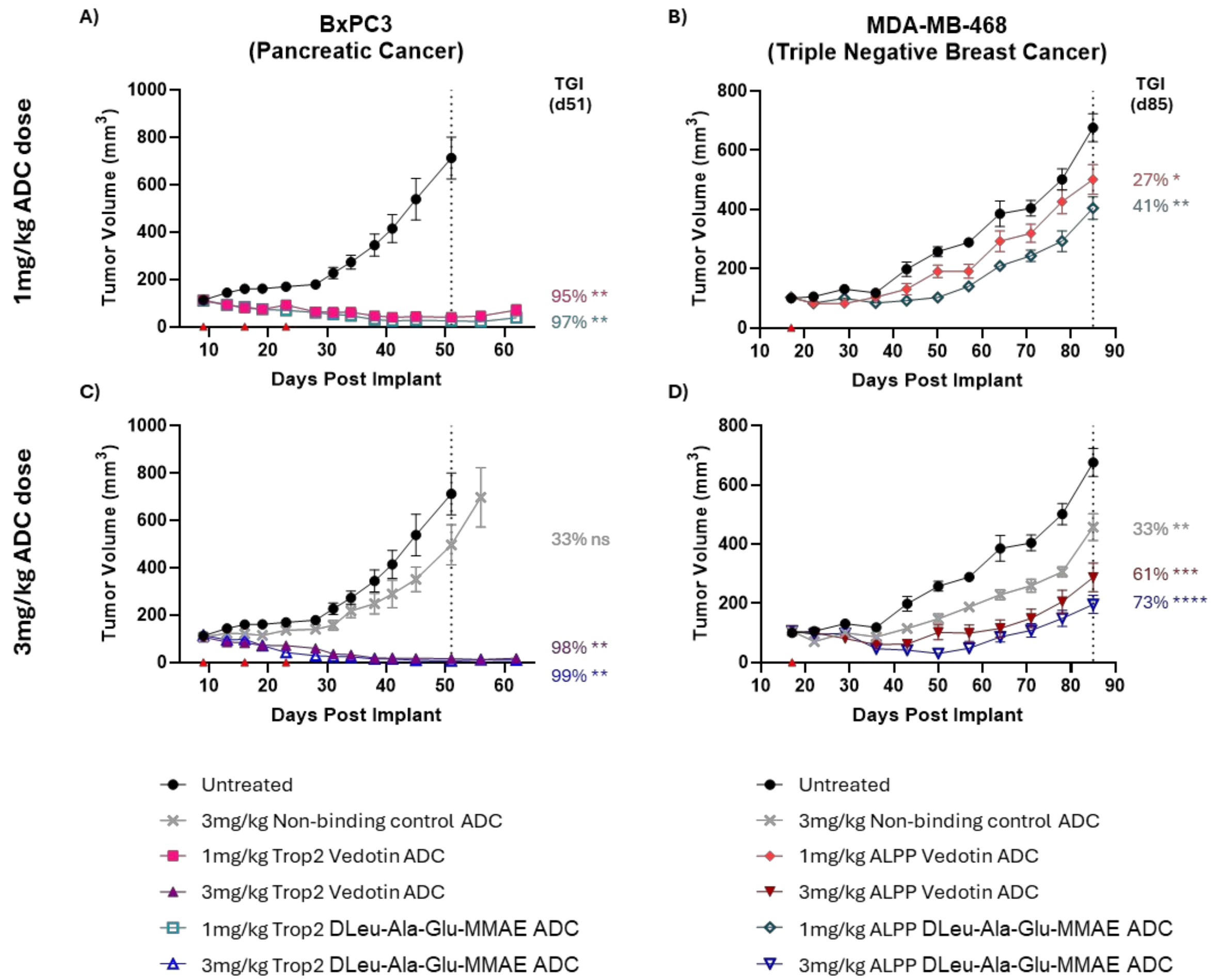

Figure S4. Comparison of the in vivo efficacy of vedotin and DLeu-Ala-Glu-MMAE ADCs in cell-line derived xenograft models of pancreatic and triple negative breast cancer. (A,C) Mean tumor volume over time of BxPC3 tumor-bearing nude mice (n=5/group). Mice were treated with 3 weekly doses of 1mg/kg (A) or 3mg/kg (C) of Trop2-vedotin, Trop2-DLeu-Ala-Glu-MMAE, or non-binding control ADC as indicated. (B,D) Mean tumor volume over time of MDA-MB-468 tumor-bearing NOD/SCID/gc KO (NSG) mice (n=6/group). Mice were treated with a single dose of 1mg/kg (B) or 3mg/kg (D) of Placental Alkaline Phosphatase (ALPP)-vedotin, ALPP-DLeu-Ala-Glu-MMAE, or non-binding control ADC as indicated. Red arrows show timing of dose administration. All ADCs were mixed 4-load ADCs. Data is presented as mean ± SEM. Statistical analysis of antitumor activity was performed with ANCOVA test. ADC, antibody-drug conjugate; TGI, percentage tumor growth inhibition compared to untreated group; *, $p<0.05$; **, $p<0.01$; ***, $p<0.001$; ****, $p<0.0001$; ns, not significant.

A)

LogD @ pH 7.4

DLeu-Ala-Glu = 1.34

LLeu-Ala-Glu = 1.34

B)

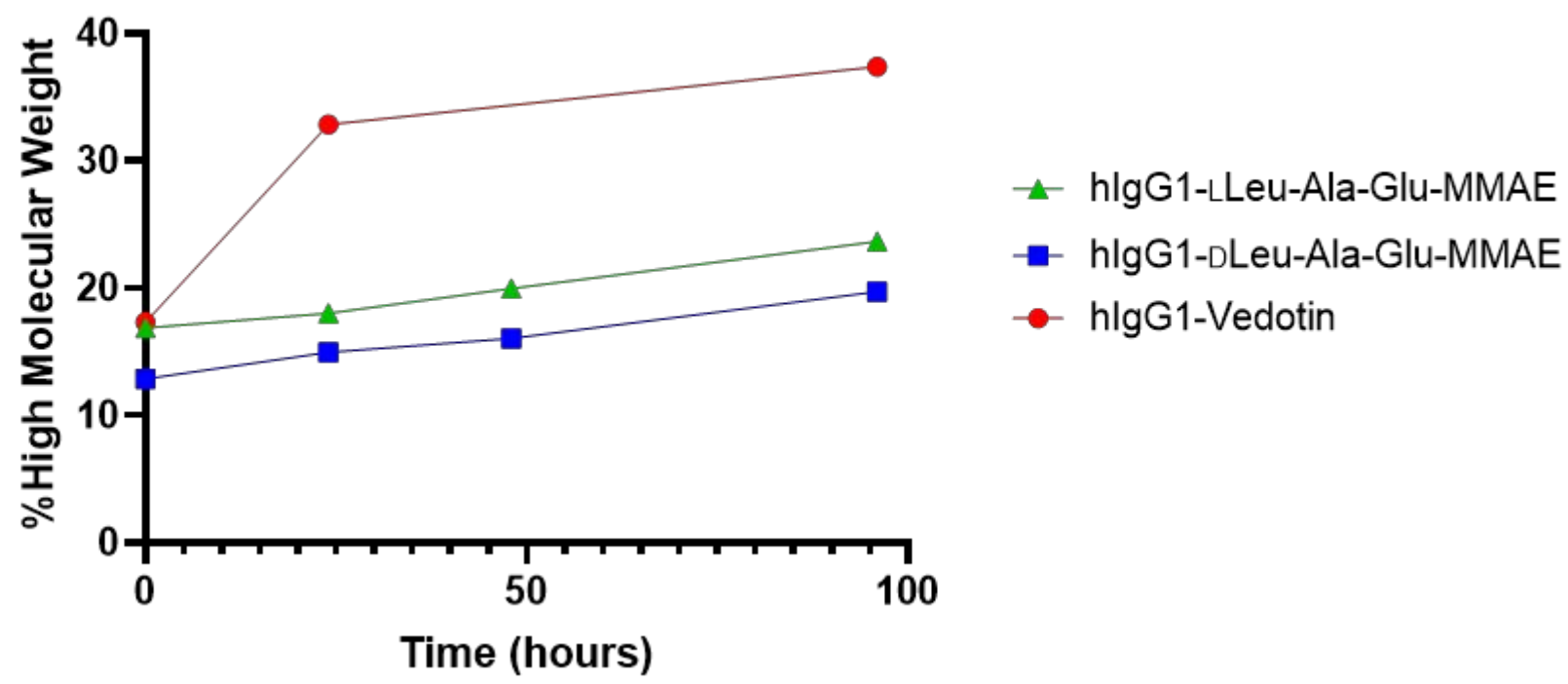

Figure S5. A) LogD @ pH 7.4 for DLeu-Ala-Glu and LLeu-Ala-Glu drug linkers. B) Non-specific binding comparing a non-targeted LLeu-Ala-Glu ADC (green) to a DLeu-Ala-Glu ADC (blue) and a vedotin ADC (red) in rat plasma.

ADC	gTab Cmax (ug/mL)	gTab AUC0-28d (d*ug/mL)	MMAE Cmax (ng/mL)	MMAE AUC (d*ng/mL)
**hIgG1-vedotin**	299 ± 29	955 ± 56	0.28 ± 0.07	0.66 ± 0.08
**hIgG1-mp-vcMMAE**	225 ± 19	738 ± 75	0.27 ± 0.08	0.76 ± 0.21
**hIgG1-DLeu-Ala-Glu**	222 ± 20	1150 ± 101	0.13 ± 0.02	0.34 ± 0.02
**hIgG1-DLeu-Leu-Met(O)**	264 ± 8.0	1070 ± 132	0.19 ± 0.04	0.51 ± 0.07
**hIgG1-DLeu-Leu-Cit**	283 ± 5.5	866 ± 133	0.26 ± 0.01	0.73 ± 0.06
**hIgG1-DLeu-Leu-Lys**	162 ± 61	535 ± 20	0.30 ± 0.17	0.72 ± 0.18
**hIgG1-Pro-Nal-Lys**	243 ± 25	487 ± 44	0.25 ± 0.03	0.60 ± 0.06
**hIgG1-Cit-Nal-Thr**	239 ± 15	525 ± 50	0.22 ± 0.03	0.56 ± 0.04

Table S1. Pharmacokinetics of hIgG1-tripeptide-MMAE ADCs after dosing a single dose in rat at 10 mg/kg (Rat-ETS1). Unnatural amino acids are D-Leucine (DLeu), Citrulline (Cit), O-Allyl-Tyrosine (Tyr(All)), and 2-Naphthylalanine (Nal).

TISSUE	Test-article Related Finding	RAT ETS1 hIgG1-tripeptide-MMAE ADCs 10 mg/kg SD	RAT ETS2 hIgG1-D-Leu-Ala-Glu-MMAE ADC 40 mg/kg SD	RAT ETS4 hIgG1-D-Leu-Ala-Glu-MMAE 10 mg/kg Q1W×4	NHP ETS1 hIgG1-D-Leu-Ala-Glu-MMAE 5 mg/kg Q1W×4
EYE	Increased corneal mitotic/apoptotic figures	Minimal	Minimal	Minimal	Absent
KIDNEY	Increased tubule mitotic/apoptotic figures	Absent	Mild	Absent	Absent
LIVER	Hepatocellular necrosis	Absent	Minimal–Moderate	Absent	Absent
LUNG	Hemorrhage	Absent	Minimal	Absent	Absent
LUNG	Increased alveolar macrophages	Absent	Absent	Minimal	Minimal
LYMPHOID ORGANS	Lymphoid depletion	Absent	Minimal–Moderate	Minimal	Mild–Moderate
SKIN	Increased mitotic/apoptotic figures	Absent	Minimal–Mild	Absent	Absent
URINARY BLADDER	Increased mitotic/apoptotic figures	Absent	Minimal–Moderate	Absent	Absent

Table S2: Summary of additional microscopic findings at terminal euthanasia 7 days post last IV dose.

ADC	acMMAE Cmax (ug/mL)	acMMAE $AUC_{0\text{-}7d}$ (d*ug/mL)	MMAE Cmax (ng/mL)	MMAE AUC (d*ng/mL)
**hIgG1-vedotin**	2260± 252	4190 ± 450	0.136 ± 0.04	0.58 ± 0.11
**hIgG1-DLeu-Ala-Glu**	2190 ± 237	3410 ± 252	0.132 ± 0.05	0.46 ± 0.11

Table S3. Pharmacokinetics of hIgG1-vedotin and hIgG1-DLeu-Ala-Glu after dosing a single dose in NHP at 5 mg/kg (NHP-ETS1).

ADC	gTab Cmax (ng/mL)	gTab AUC_{0-last} (d*ng/mL)	MMAE Cmax (ng/mL)	MMAE AUC (d*ng/mL)
**hIgG1-vedotin**	299 ± 29	955 ± 56	0.28 ± 0.07	0.66 ± 0.08
**hIgG1-DLeu-Ala-Glu**	265 ± 2.7	1197 ± 129	0.17 ± 0.01	0.45 ± 0.04
**hIgG1-LLeu-Ala-Glu**	312 ± 9.5	1447 ± 68.6	0.24 ± 0.01	0.61 ± 0.07

Table S4. Pharmacokinetics of hIgG1-vedotin, hIgG1-DLeu-Ala-Glu and hIgG1-LLeu-Ala-Glu after dosing a single dose in rat at 10 mg/kg.

Supplemental Chemical and Analytical Data

Chemical Syntheses

Preparation of p-azido-benzyl alcohol (Az-PABA)

To a round bottomed flask was added p-amino-benzyl alcohol (100 mol%) suspended in 5 M HCl (5 mL per g PABA). The flask was cooled to 4 ^{o}C followed by the dropwise addition of aqueous $NaNO_2$ (150 mol%; 20 mL per gram PABA). NaN_3 was then added and the reaction was warmed to room temperature and incubated for 16 h. The reaction was diluted in saturated $NaHCO_3$ and extracted with EtOAc. The extract was dried with $MgSO_4$ and concentrated. The product was purified using an EtOAc/hexanes gradient (6%-42% EtOAc) with a SNAP-KP-Sil Biotage column yielding the title compound as an orange material (90% yield). ^{1}H-NMR (d_6-DMSO) δ 7.38-7.35 (C=CH, d, 2H), 7.11-7.07 (C=CH, d, 2H), 5.25-5.22 (OH, m, 1H), 4.50-4.46 (CH2, d, 2H)

Preparation of p-azido-benzyl bromide.

To a round bottomed flask was added Az-PABA (100 mol%) dissolved in chloroform under a nitrogen atmosphere. To the solution was added PBr_3 (120 mol%) dropwise. The reaction was incubated for 2 h at which point it was diluted with $CHCl_3$ and washed with 1 M HCl followed by brine. The extract was dried with $MgSO_4$ and concentrated. The product was purified using an EtOAc/hexanes gradient (6%-42% EtOAc) with a SNAP-KP-Sil Biotage column to provide the title compound in 75% yield.

Preparation of methyl (2-(7-hydroxy-2-oxo-2*H*-chromen-4-yl)acetyl)glycinate (HO-Coum-Gly-OMe).

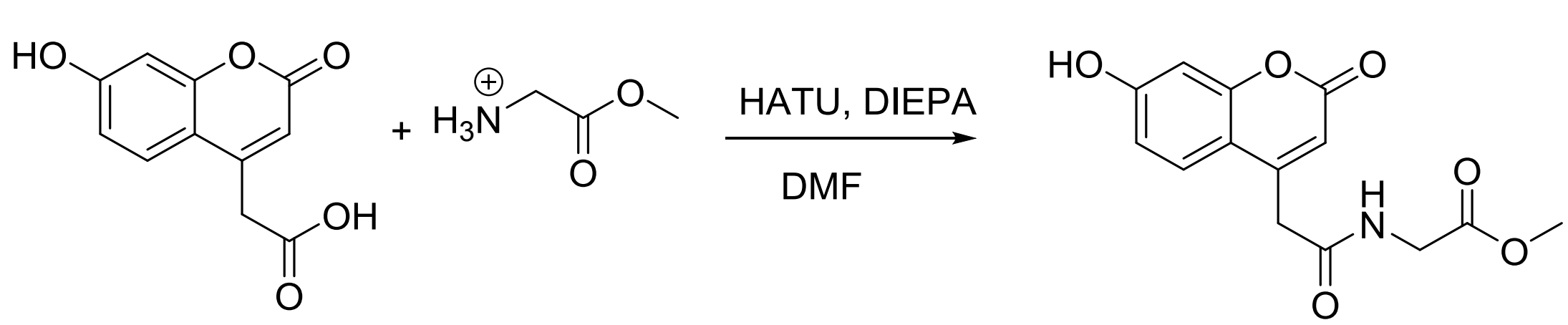

To a scintillation vial was added H-Gly-OMe (300 mol%) dissolved in DMF and DIPEA (350 mol%). To this vial was added 2-(6-hydroxy-2-oxo-2*H*-chromen-4-yl)acetic acid (100 mol%). DMF was then added until both reagents were fully dissolved. HATU (110 mol%) was then added followed by DIPEA (110 mol%) and the reaction was stirred for 45 min. At that time, the reaction was diluted in EtOAc and washed with 200 mM HCl. The aqueous layer was back extracted 3x with EtOAc. The combined organics were washed with brine, dried with $MgSO_4$, and concentrated to provide the title compound, which was purified in 80% yield from boiling isopropyl alcohol.

Preparation of methyl (2-(7-((4-azidobenzyl)oxy)-2-oxo-2*H*-chromen-4-yl)acetyl)glycinate (Az-PABE-Coum-Gly-OMe)

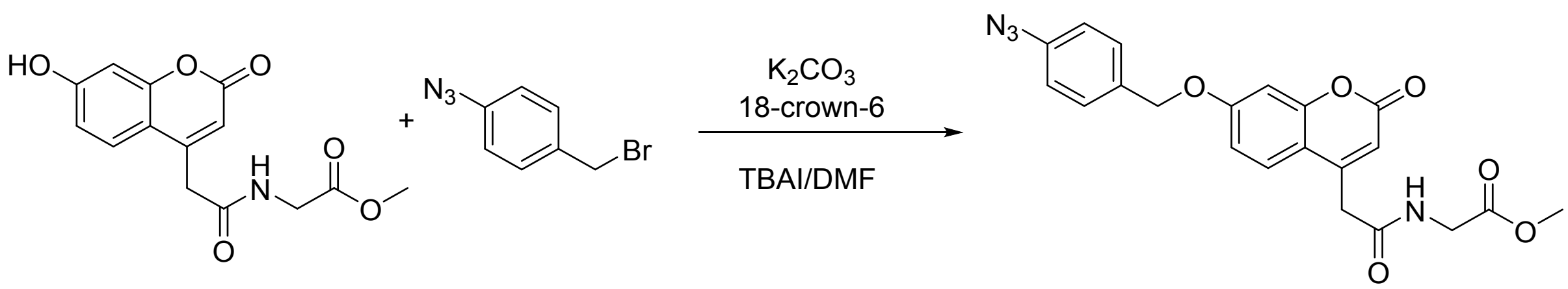

To a round bottomed flask was added HO-Coum-Gly-OMe (300 mol%), K_2CO_3 (150 mol%), and 18-crown-6 ether (200 mol%) suspended in DMF. After 15 min of vigorous stirring, Az-PAB-Br (100 mol%) was added slowly in 4 separate aliquots. To the resulting solution was added tetrabutylammonium iodide (15 mol%), which was then stirred for 16 h. At that point the reaction was diluted into EtOAc and washed with 200 mM HCl and brine. The separated organic layer was dried with $MgSO_4$ and concentrated to provide the title compound as crude material that was used without further purification.

Preparation of (2-(7-((4-azidobenzyl)oxy)-2-oxo-2H-chromen-4-yl)acetyl)glycine (Az-PABE-Coum-Gly-OH)

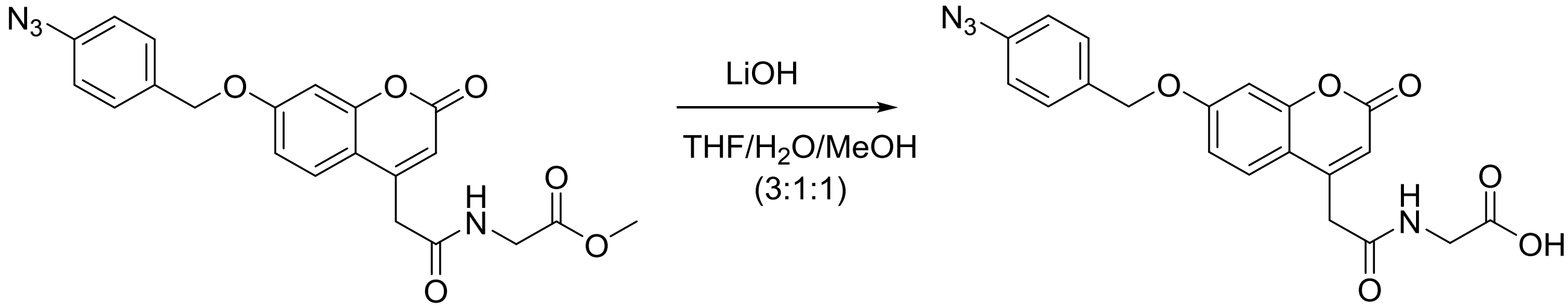

To a round bottomed flask was added crude Az-PABE-Coum-Gly-OMe (100 mol%) in THF (20 mL per 500 mg). To the vial was added MeOH (6 mL per 500 mg) and H_2O (6 mL per 500 mg). At that time LiOH (200 mol%) was added and the reaction was stirred for 1 h whereupon the reaction was diluted with EtOAc and washed twice with 200 mM HCl. The separated organic layer was dried with $MgSO_4$ and concentrated to provide the title compound in 88% yield. ^{1}H-NMR (d_7-DMF) δ 8.80 (NH, t, 1H), 8.03-8.01 (C=CH, d, 1H), 7.80-7.77 (C=CH, d, 2H), 7.38-7.36 (C=CH, d, 2H), 7.25 (C=CH, s, 1H), 7.24-7.20 (C=CH, d, 1H), 6.58 (C=CH, s, 1H), 5.47 (CH2, s, 2H), 4.14 (CH2, d, 2H), 4.08 (CH2, s, 2H).

Preparation of P1-PABE-Coum-Gly-OH where P1 = Fmoc-Leu-OH, Fmoc-D-Leu-OH, Fmoc-Ala-OH, Fmoc-Met-OH, Fmoc-Pro-OH, Fmoc-Cit-OH, Fmoc-Nal-OH, Fmoc-Tyr(All)-OH, Fmoc-Phe-OH, Fmoc-Lys(Mtt)-OH, Fmoc-Thr(Trt)-OH, Fmoc-Glu(O-2-PhiPr)-OH, wherein Cit is citrulline, and Nal is alanine in which its methyl side chain is substituted by napthth-1-yl.

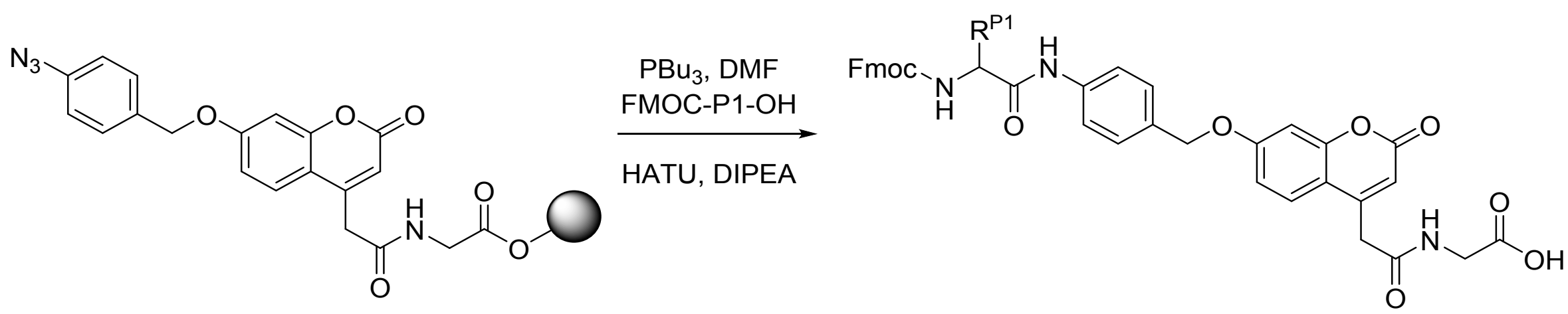

To resin (2-chloro-trityl chloride or rink acid; 100 mol%) swollen in dry DCM was added Az-PABE-Coum-Gly-OH (300 mol%) and DIPEA (310 mol%) dissolved in dry DCM. After mixing for 2 h, the solution was expelled and resin was washed with DCM. To an open round-bottomed flask was added Az-PABE-Coum-Gly-O-linked resin swollen in DMF followed by the addition of PBu_3 (250 mol%) and DIPEA (250 mol%). After mixing for 2 h, the solution was expelled and resin was washed with DMF, DCM, and Et_2O and dried overnight under vacuum. To a vial was added Fmoc-P1-OH (600 mol%) and HATU (600 mol%) dissolved in DMF, followed by DIPEA (800 mol%). The mixture was vortexed for 1 min and then added to the previously synthesized PBu_3 activated Az-PABE-Coum-Gly-O-linked resin (rink acid resin for Fmoc-Lys(Trt)-OH, Fmoc-Thr(Trt)-OH, and Fmoc-Glu(O-2-PhiPr), 2-chloro-trityl resin for all other amino acids) swollen in DMF. After mixing for 2 h, the solution was expelled and resin was washed with DMF and DCM. Fmoc-P1-PABE-Coum-Gly-OH was cleaved from

resin using 0.2% TFA in DCM (for rink acid resin) or 5% TFA in DCM (for 2-chloro trityl resin) and purified by RP-HPLC.

FMOC chemistry was used to prepare the Gly-D-Lys-Gly-Gly moiety, covalently attached to the cellulose solid support, in which the cellulose hydroxyl groups of the laser-perforated cellulose paper were first modified as glycine esters. The FMOC group was then removed to provide a free amine, which was confirmed by a pH sensitive indicator. The next amino acid was added, and the process was repeated. For compatibility with a 96-well microtiter plate, the laser perforated discs were 6 mm in diameter to which are added 1 μL aliquots of the FMOC protected amino acid solutions.

FMOC-P1-PABE-Coum-Gly-OH was then attached to the free amino group of the NH_2- Gly-D-Lys-Gly-Gly- residue. The key step in the reaction sequence is reduction of the resin-bound azide intermediate, which provides an iminophosphorane intermediate that is stable enough towards self-immolation in order for the coupling reaction with the first incoming FMOC-amino acid to occur. The P2 and P3 amino acids were then added by standard FMOC chemistry followed by acylation of the free amino group of the deprotected P3 residue to provide the resin-bound library compound, which was cleaved from the resin using an ammonia gas chamber. In Scheme 1, R^{P1}, R^{P2} and R^{P3} are the amino acid side chains of the P1, P2 and P3 amino acid residues, respectively, and X represents the other amino acids in the NH_2-Gly-Gly-D-Lys-Gly-Gly- pentapeptide that tethers the fluorescently labelled tripeptide to the cellulose solid support.

Scheme 1. Preparation of Fluorescently labelled library compounds

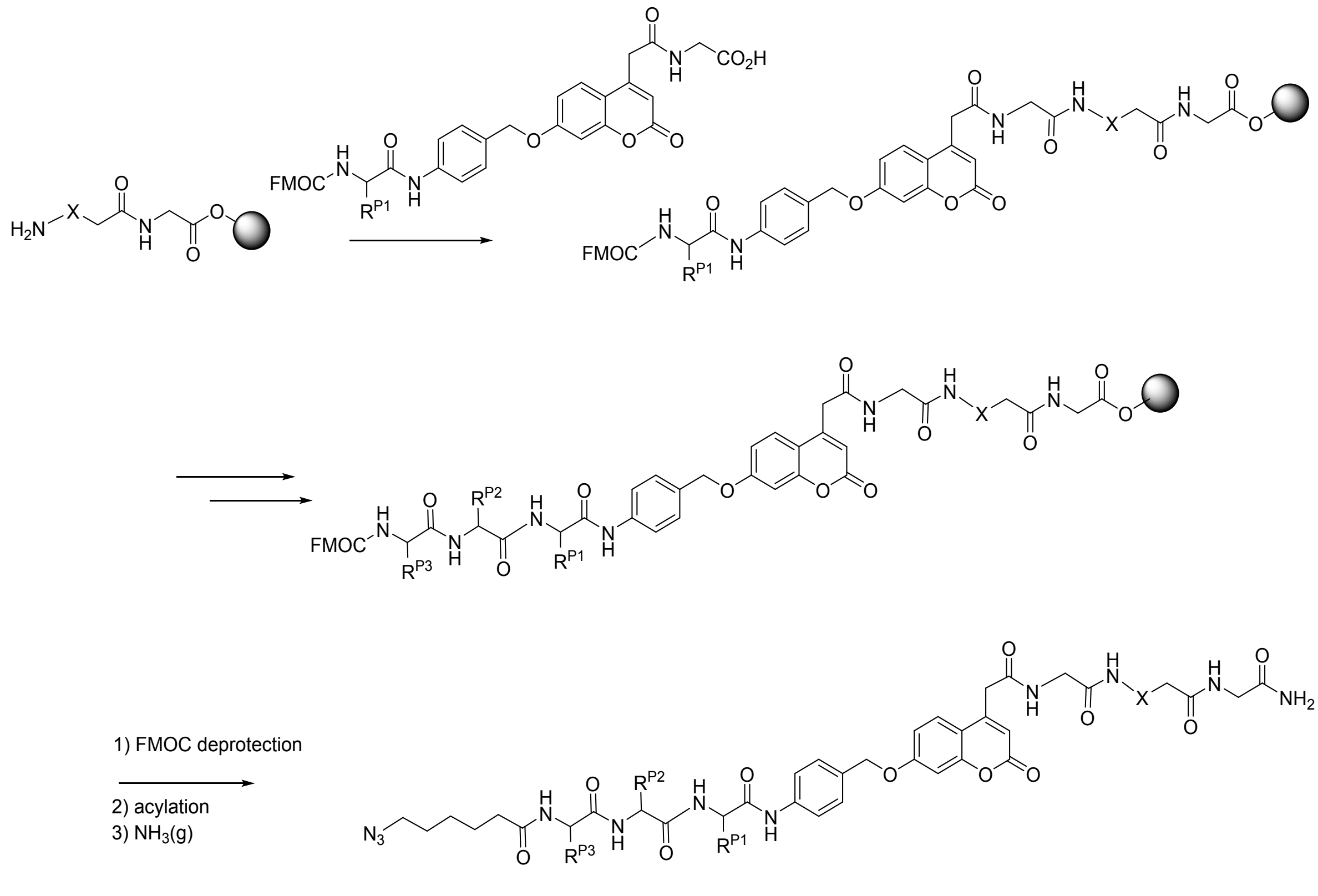

Preparation of Drug Linker compounds.

Drug Linker compounds in which MMAE is the Drug Unit and which were used for preparing the selected subset of Drug Conjugates, are represented by the following structure.

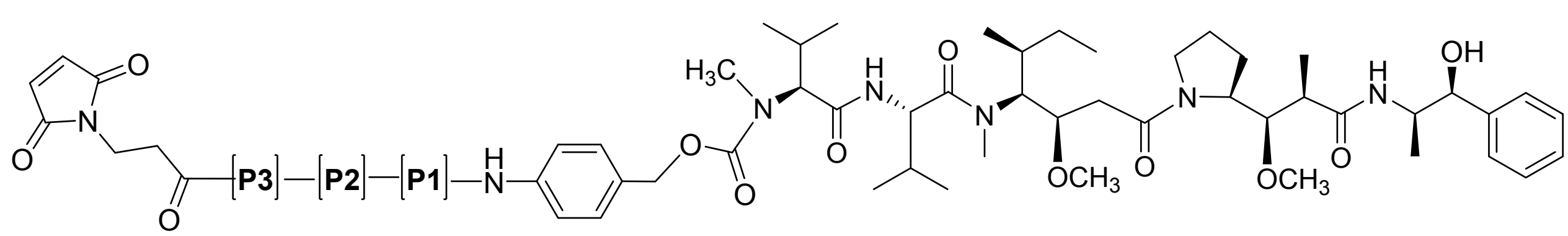

mp-[P3]-[P2]-[P1]-PABC-MMAE

Preparation of Resin-bound MMAE:

Resin-bound MMAE was prepared using DHP HM functionalized resin according to the procedure of Scheme 2A.

<u>Scheme 2A</u>. Preparation of resin bound MMAE

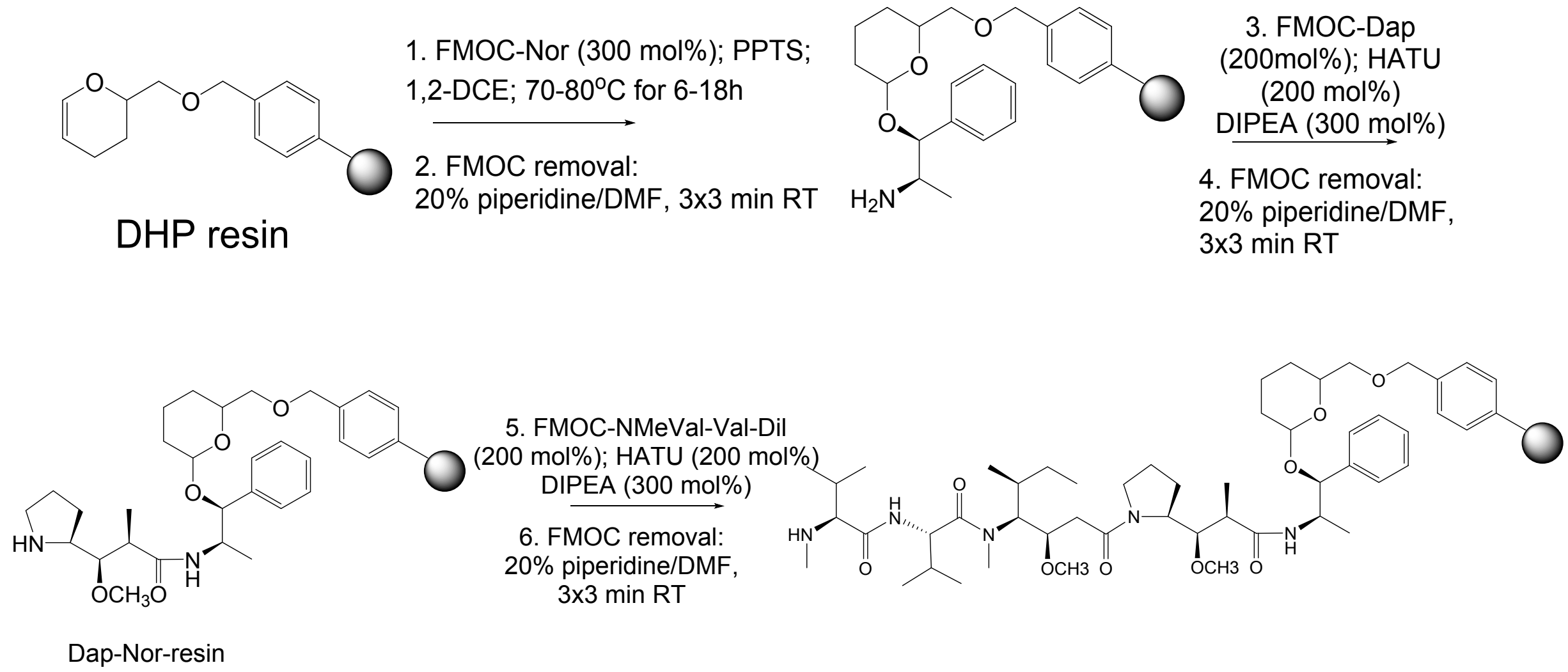

Briefly, to synthesize MMAE on resin, FMOC-Norephedrine and pyridinium p-toluenesulfonate (PPTS) were dissolved in dichloroethane, added to DHP HM functionalized resin, and incubated at 70 °C for 8 h. After deprotection, FMOC-Dap was subsequently activated with HATU and DIPEA and then added to the norepinephrine-resin material. The reaction sequence was repeated with FMOC-N-MeVal-Val-Dil, which after deprotection, provided resin-bound MMAE.

Preparation of tripeptide based MMAE Drug Linker compounds.

Tripeptide-based Drug Linker compounds in which the Drug Unit is derived from MMAE and having tripeptide sequences selected from Tables 1A and 1B were prepared from resin-bound MMAE according to the procedures of Scheme 3.

Scheme 3. Preparation of tripeptide-based Drug Linker compounds from resin-bound MMAE

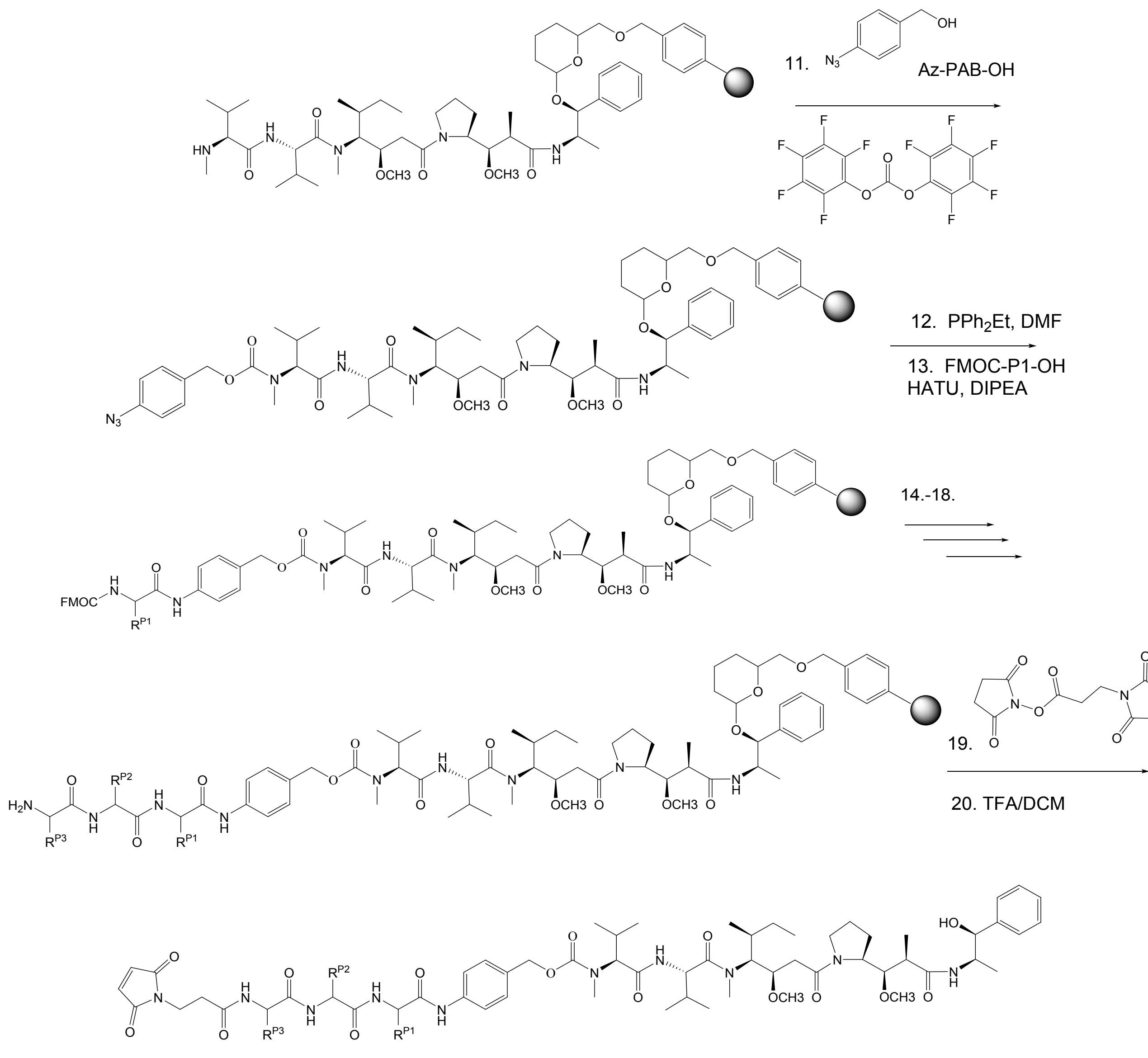

Briefly, Az-PAB-OH, prepared by reaction of NaN_3 with the diazonium salt from p-aminobenzyl alcohol and $NaNO_2$ in 5M HCl, was reacted with bis(pentafluorophenyl) carbonate and added to MMAE on resin. The azido group of Az-PABC-MMAE was then reduced to the iminophosphorane with PPh_2Et, followed by addition of FMOC-P1. After deprotection, amino acids P2 and P3 were then added through conventional FMOC peptide chemistry followed by reaction of the activated ester 3-(Maleimido)propionic acid N-hydroxysuccinimide ester with the deprotected amine of the terminal P3 amino acid. After

cleavage from resin using TFA in DCM, the Drug Linker compound so obtained was purified by reverse phase HPLC.

UPLC-MS was performed on a Waters single quad detector mass spectrometer interfaced to a Waters Acquity™ UPLC system using a UPLC method wherein Solvent A is 0.1 % aqueous formic acid and Solvent B - acetonitrile with 0.1% formic acid.

<u>Table 2</u>. UPLC-MS data for MMAE Drug Linker compounds

Tripeptide* Sequence	Molecular Formula	MS Calc. $(M+H)^+$	MS found
D-Leu-Leu-Met(O)	$C_{71}H_{110}N_{10}O_{16}S$	1391.8	1392.0
D-Leu-Ala-Glu	$C_{68}H_{102}N_{10}O_{17}$	1331.7	1332.0
D-Leu-Leu-Cit	$C_{72}H_{112}N_{12}O_{16}$	1401.8	1402.0
Leu-Ala-Glu	$C_{68}H_{102}N_{10}O_{17}$	1330.7	1332.2
D-Leu-Ala-Cit	$C_{69}H_{106}N_{12}O_{16}$	1358.8	1359.3
Pro-Leu-Glu	$C_{70}H_{104}N_{10}O_{17}$	1356.8	1357.4
Thr-Glu-Leu	$C_{69}H_{104}N_{10}O_{18}$	1360.8	1361.4
D-Leu-Glu-Cit	$C_{71}H_{108}N_{12}O_{18}$	1416.8	1417.4
Glu-Pro-Cit	$C_{70}H_{104}N_{12}O_{18}$	1400.8	1402.2
O-allyl-Tyr-Pro-Cit	$C_{77}H_{110}N_{12}O_{17}$	1474.8	1475.9
Lys-Leu-Cit	$C_{72}H_{113}N_{13}O_{16}$	1415.8	1417.6
O-allyl-Tyr-DLeu-Glu	$C_{77}H_{110}N_{10}O_{18}$	1462.8	1463.8
D-Leu-Ala-Gln	$C_{68}H_{103}N_{11}O_{16}$	1331.6	1331.4
D-Leu-Ala-(Se-Met)	$C_{68}H_{104}N_{10}O_{15}Se$	1381.6	1381.4
D-Leu-Glu-Ala	$C_{68}H_{102}N_{10}O_{17}$	1332.6	1332.2
D-Leu-Ala-Ala	$C_{66}H_{100}N_{10}O_{15}$	1274.6	1275.0
D-Leu-Ala-Met(O)	$C_{68}H_{104}N_{10}O_{16}S$	1350.7	1350.1
D-Leu-Leu-Glu	$C_{71}H_{108}N_{10}O_{17}$	1374.7	1373.9
D-Ala-Ala-Glu	$C_{65}H_{96}N_{10}O_{17}$	1290.5	1290.3
Ala-Ser-Glu	$C_{65}H_{96}N_{10}O_{18}$	1306.5	1306.4
D-Leu-Ala-Asp	$C_{67}H_{100}N_{10}O_{17}$	1318.6	1318.5
D-Leu-Val-Gln	$C_{70}H_{107}N_{11}O_{16}$	1359.7	1359.9

D-Leu-Ala-Gla	$C_{69}H_{102}N_{10}O_{19}$	1376.6	1376.5
D-Leu-Ala-Lys	$C_{69}H_{107}N_{11}O_{15}$	1332.7	1331.5
Ala-Ser-Asp	$C_{64}H_{94}N_{10}O_{18}$	1292.5	1292.3
Val-Gln-Glu	$C_{69}H_{103}N_{11}O_{18}$	1375.6	1375.5
Ala-Ser-Pro	$C_{65}H_{96}N_{10}O_{16}$	1272.7	1274.6
Pro-Ser-Ser	$C_{65}H_{96}N_{10}O_{17}$	1289.7	1290.0
Gln-Ser-Ala	$C_{65}H_{97}N_{11}O_{17}$	1303.7	1305.3
Ser-Ser-Pro	$C_{65}H_{96}N_{10}O_{17}$	1289.7	1290.4
Glu-Ser-Ala	$C_{65}H_{96}N_{10}O_{18}$	1304.7	1306.2
Ser-Asn-Asn	$C_{65}H_{96}N_{12}O_{18}$	1332.7	1334.4
D-Ser-Ser-Glu	$C_{65}H_{96}N_{10}O_{19}$	1320.7	1322.4

*Abbreviations: Aib = α-aminoisobutyric acid, Cit = citrulline, Met(O) = methionine sulfoxide, Nal = naphthal-1-yl alanine, (Se-Met) = Selenomethionine, Gla = gamma-carboxyglutamate

Structures of the tripeptide-based Drug Linker compounds of Table 2, as well as the comparator dipeptide-based Drug Linker compound vedotin are as follows:

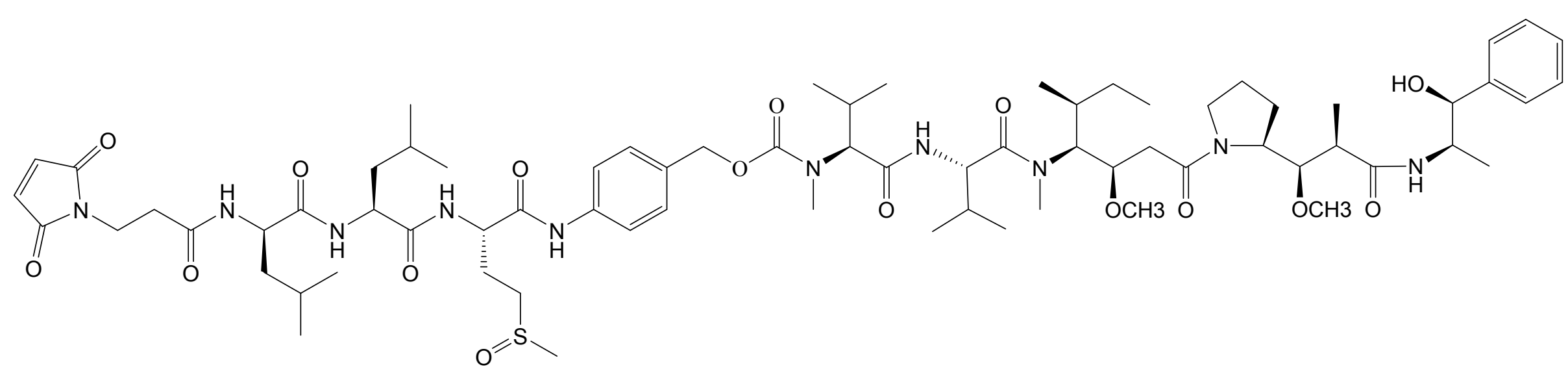

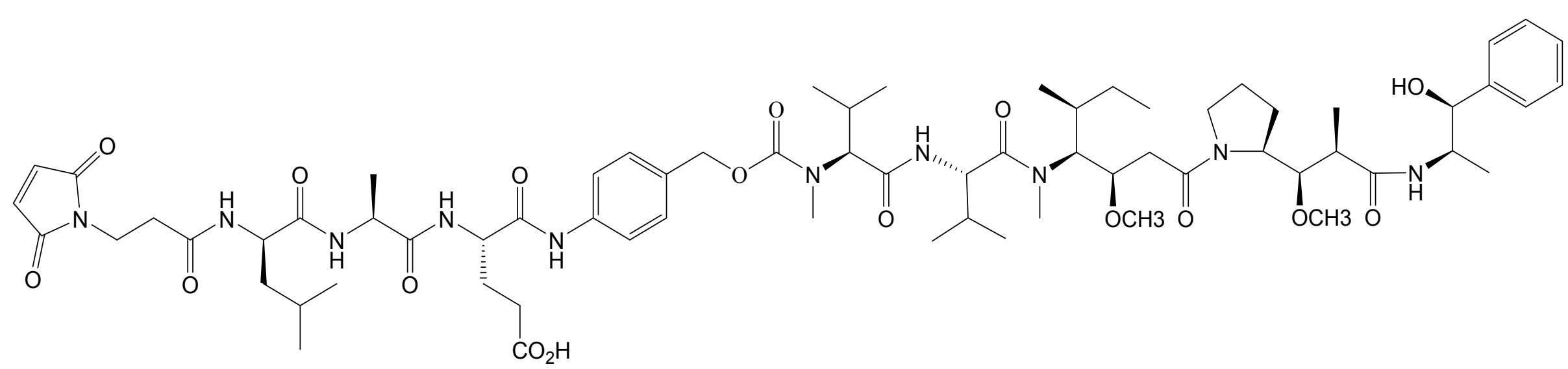

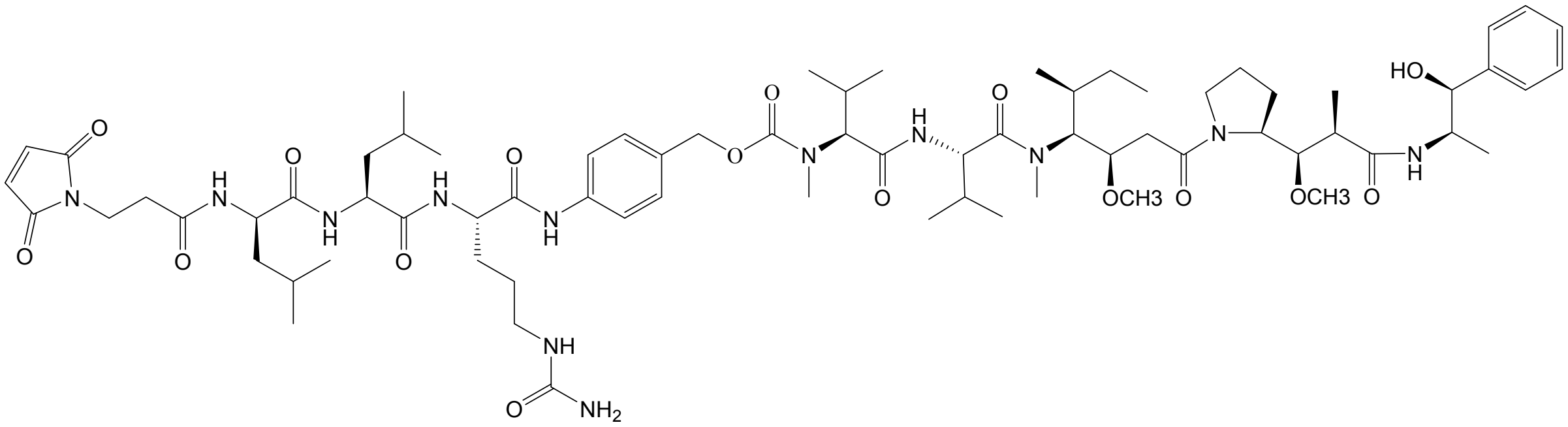

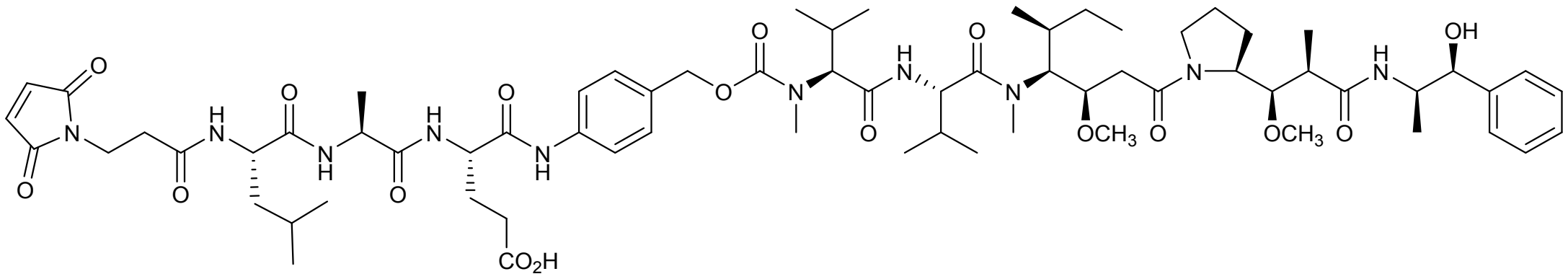

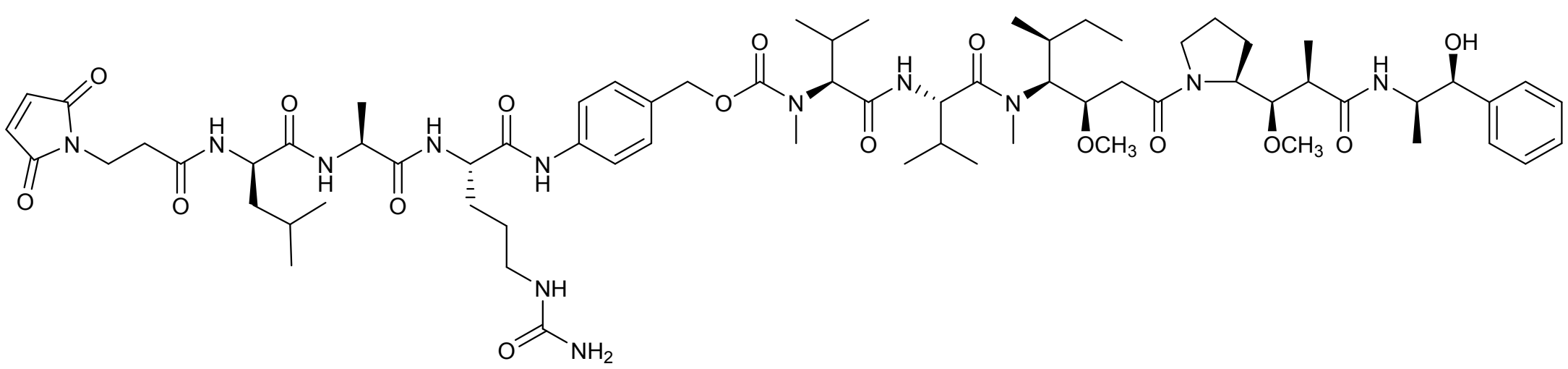

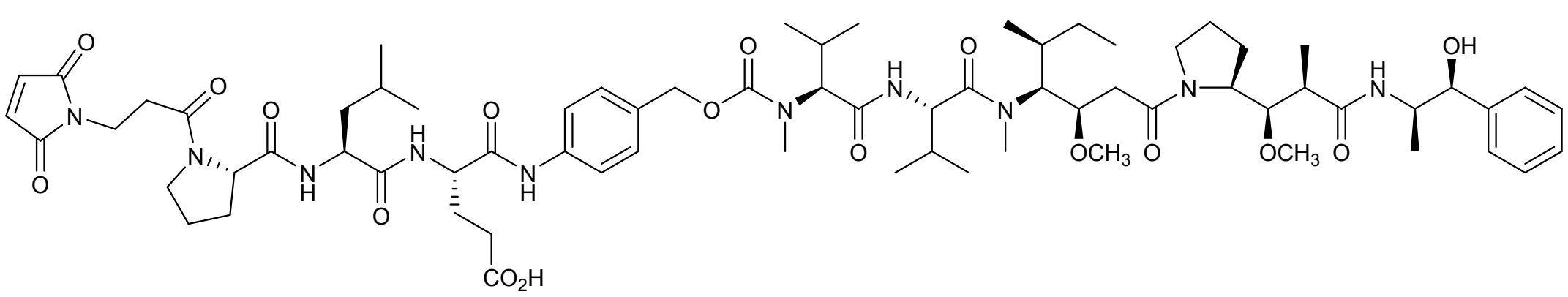

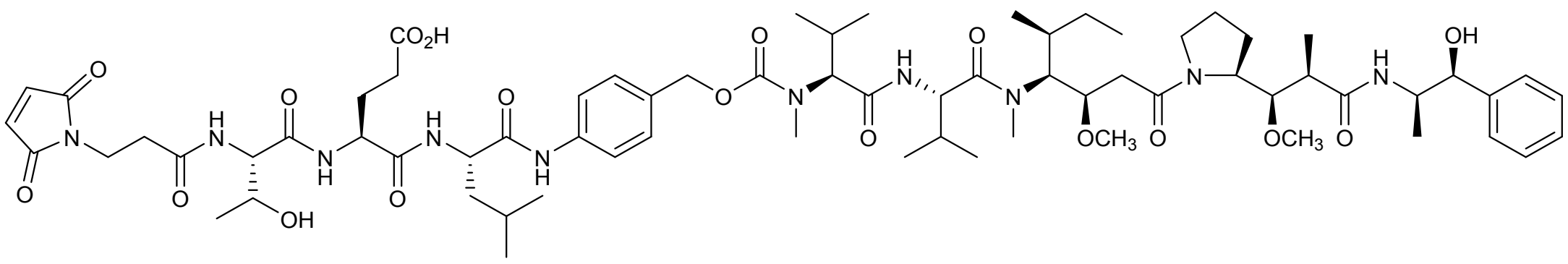

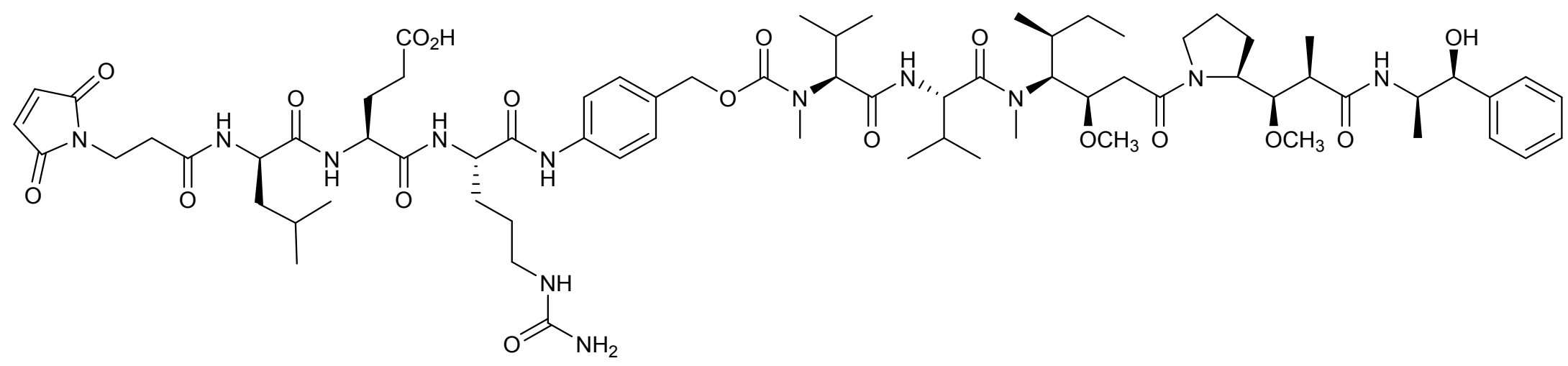

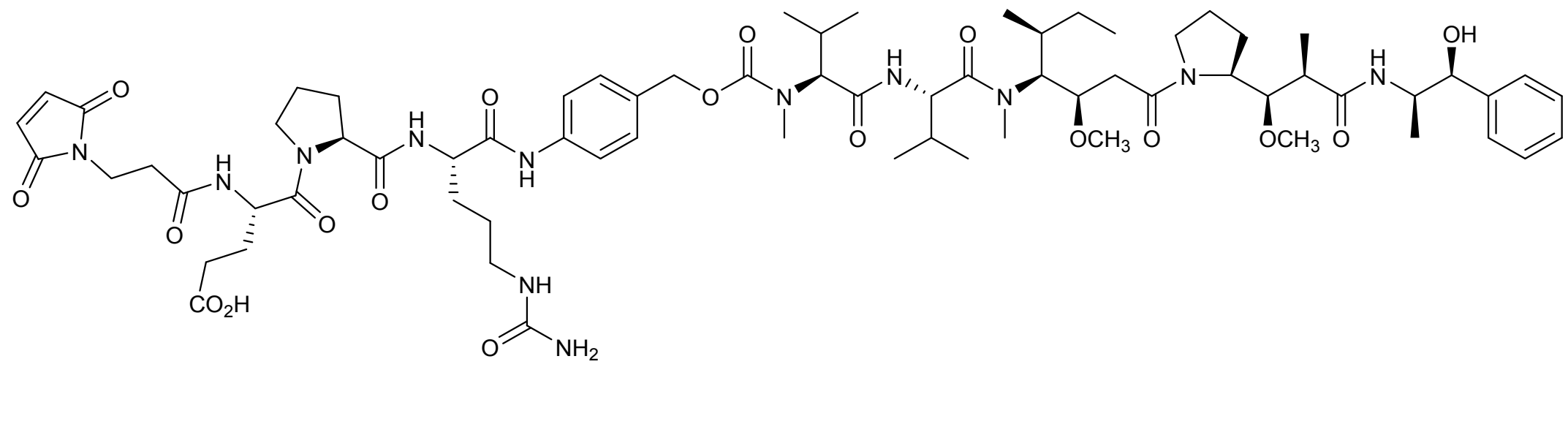

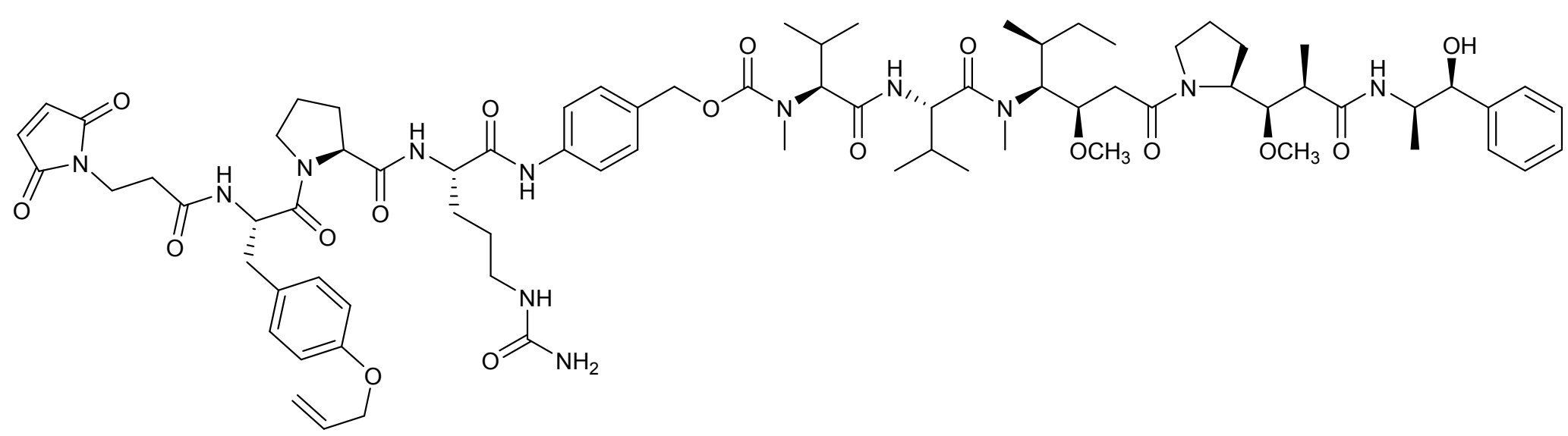

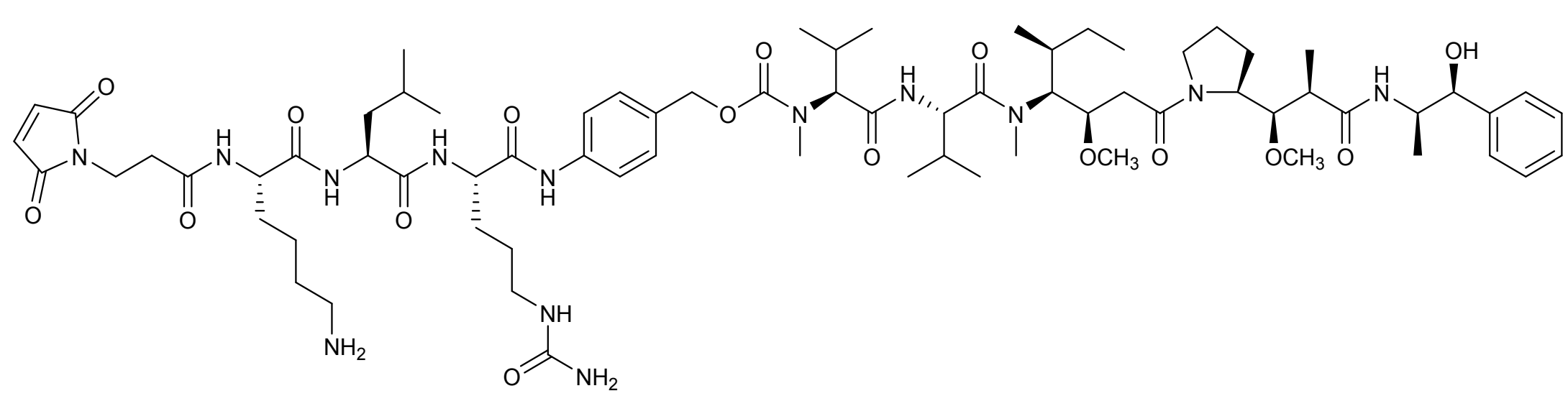

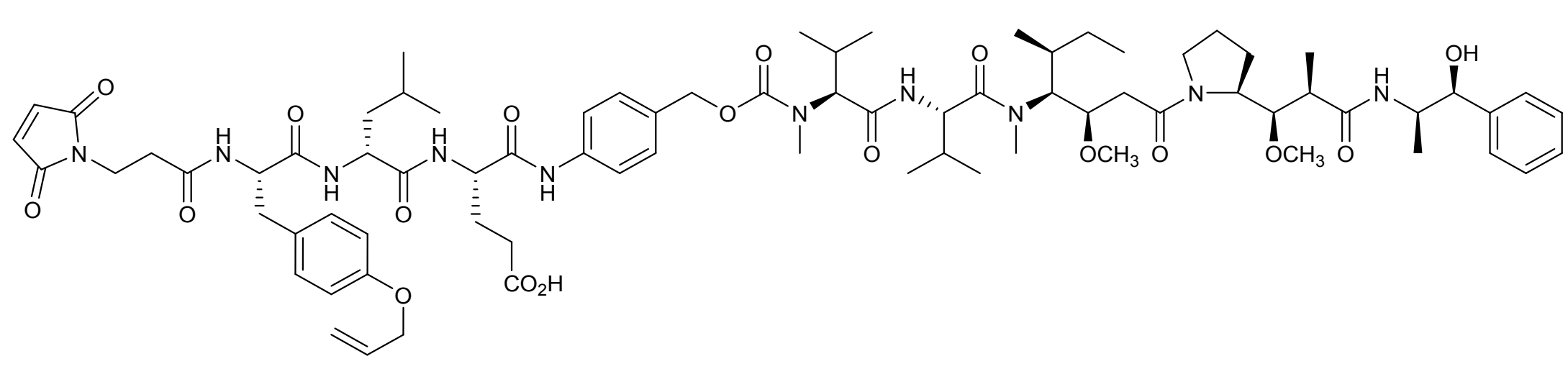

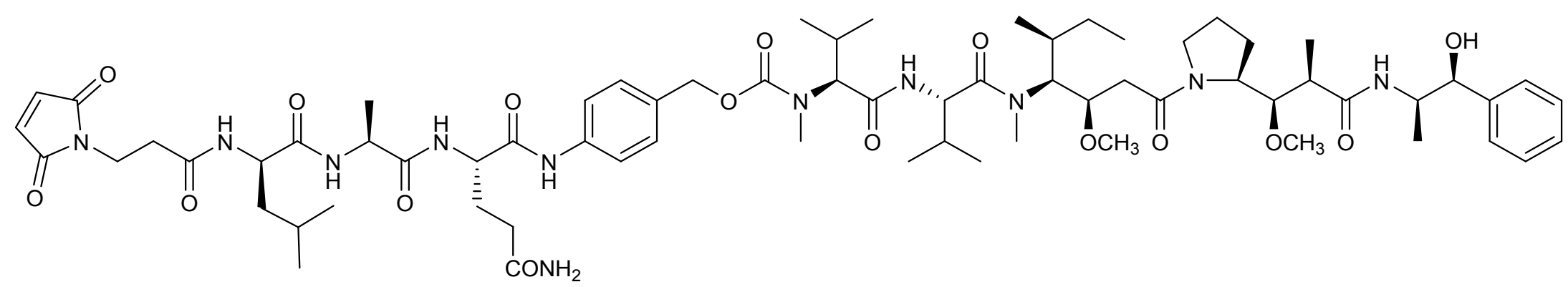

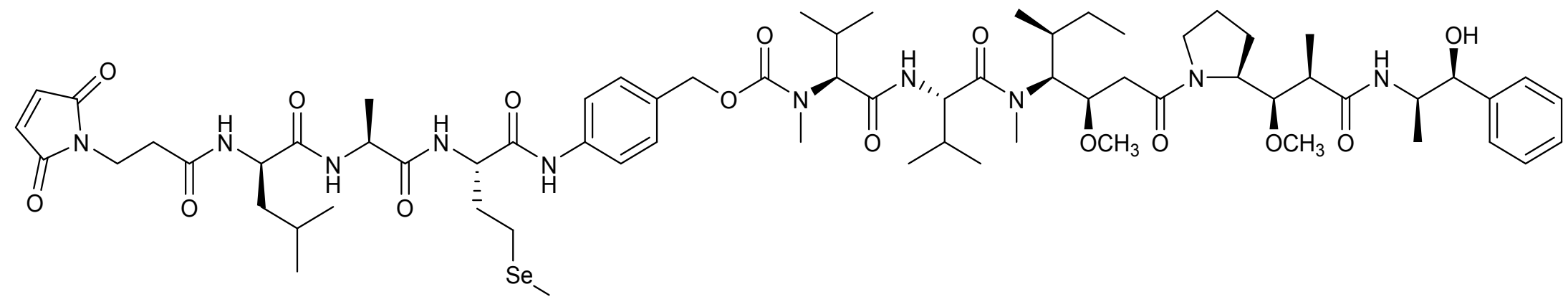

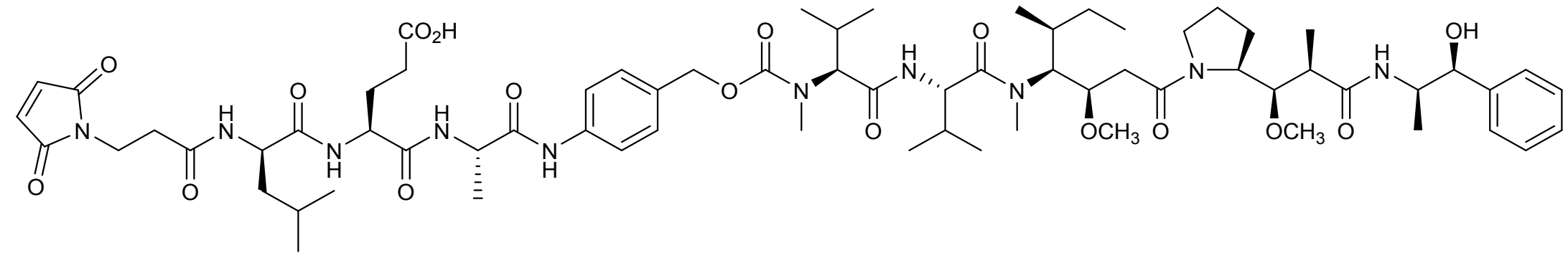

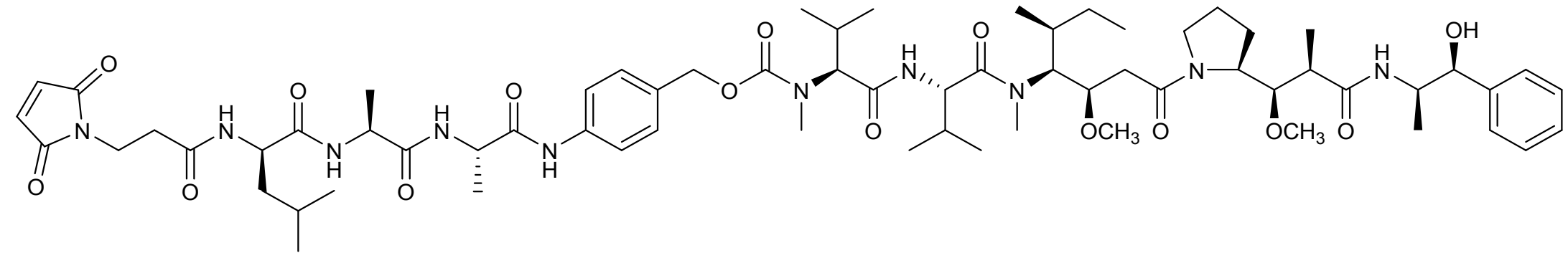

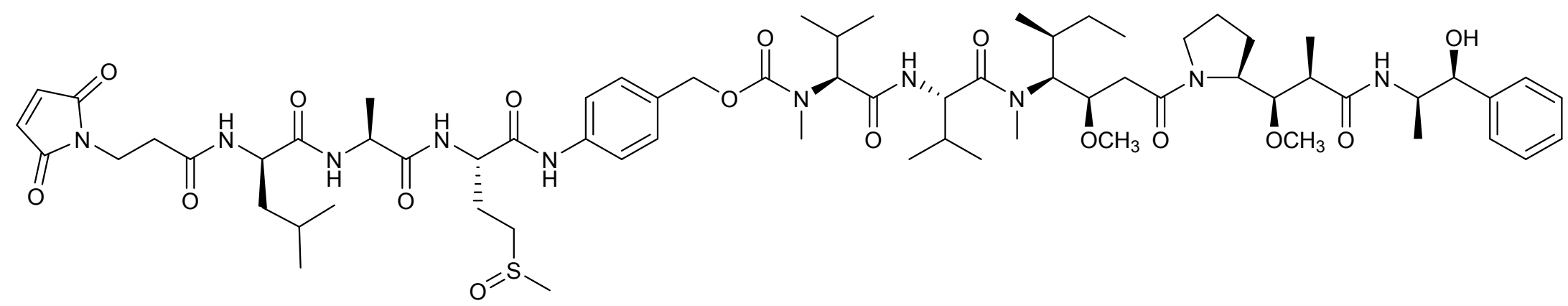

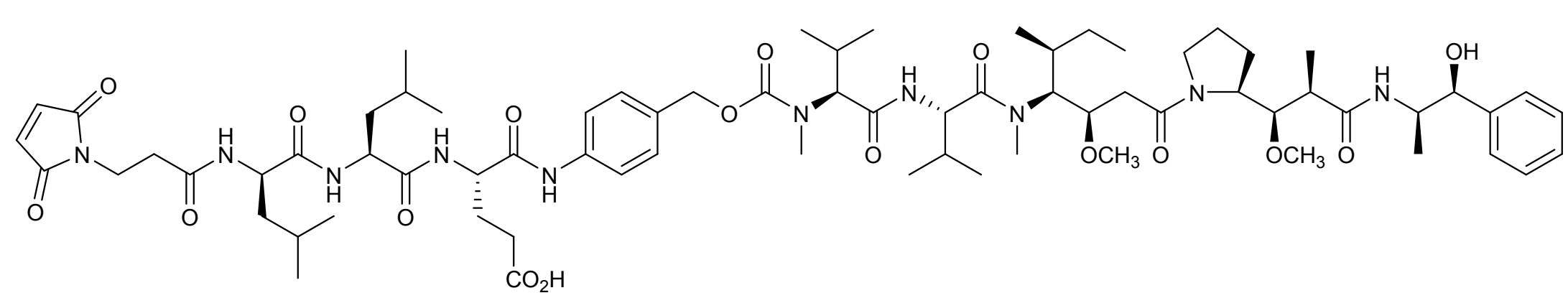

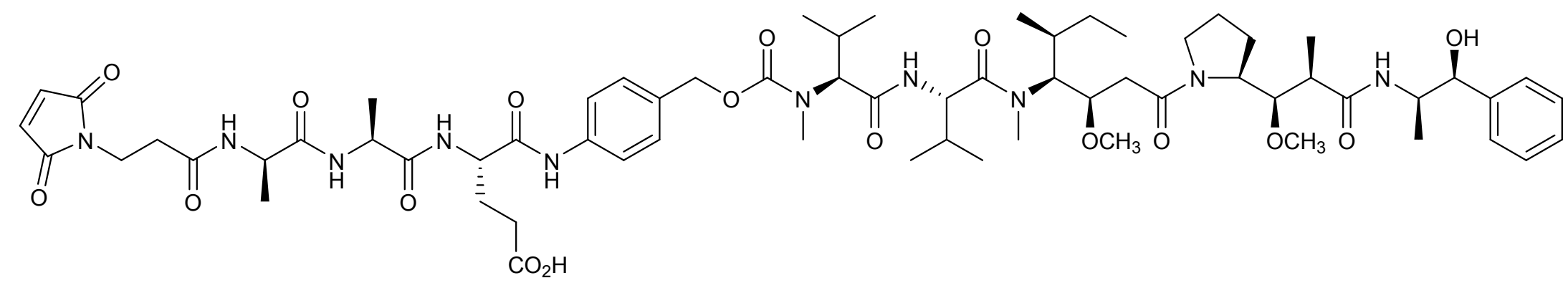

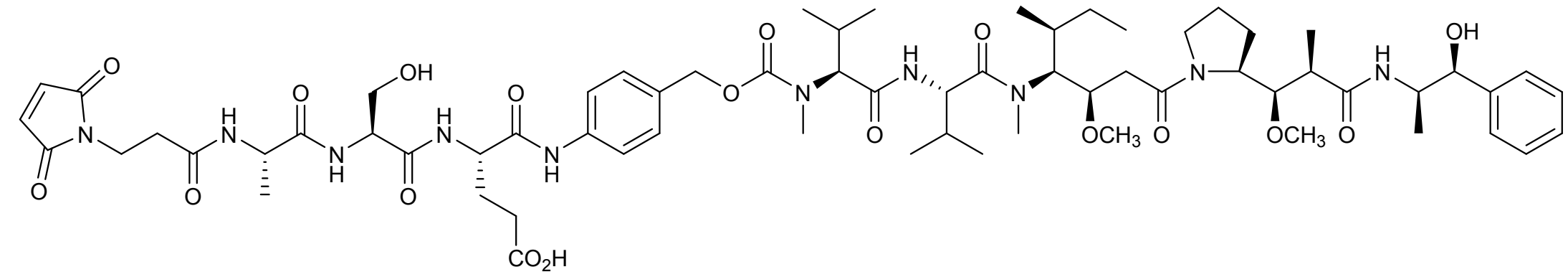

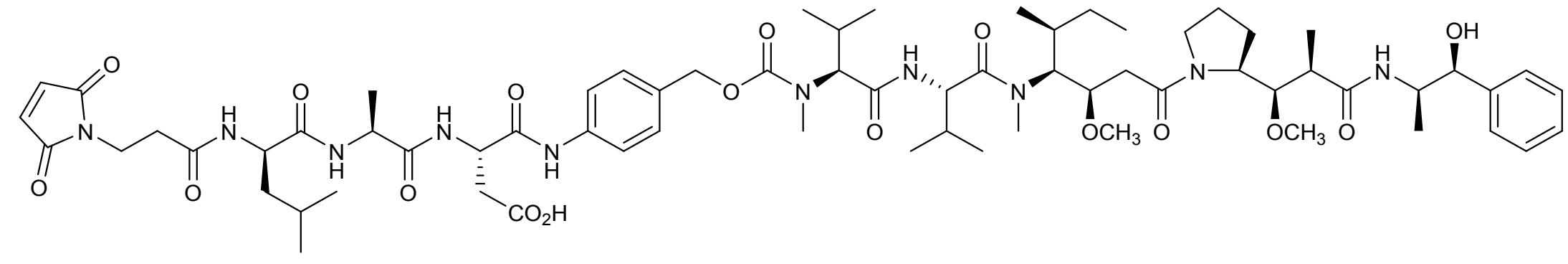

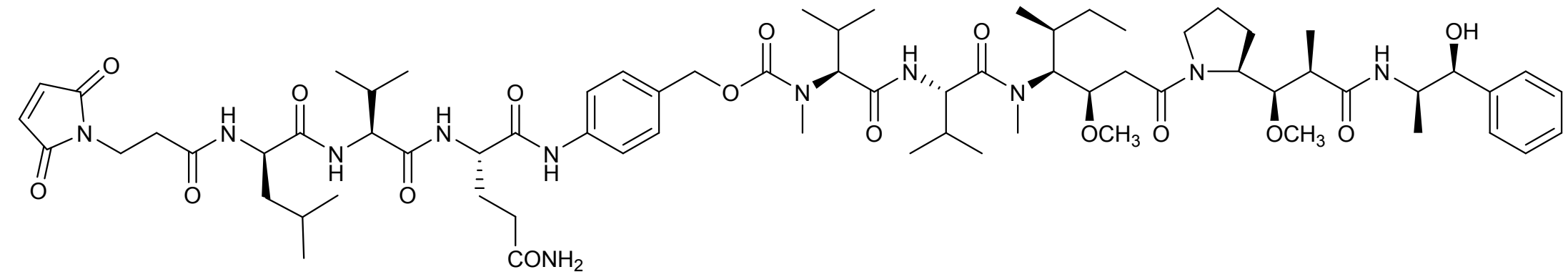

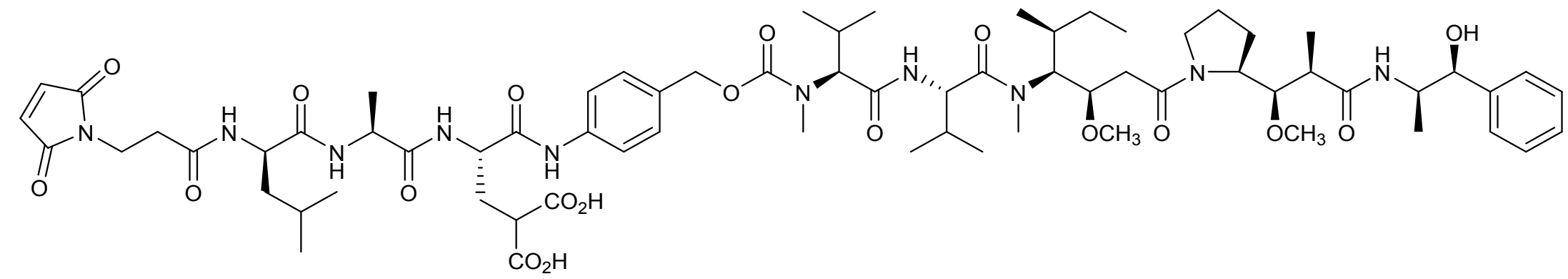

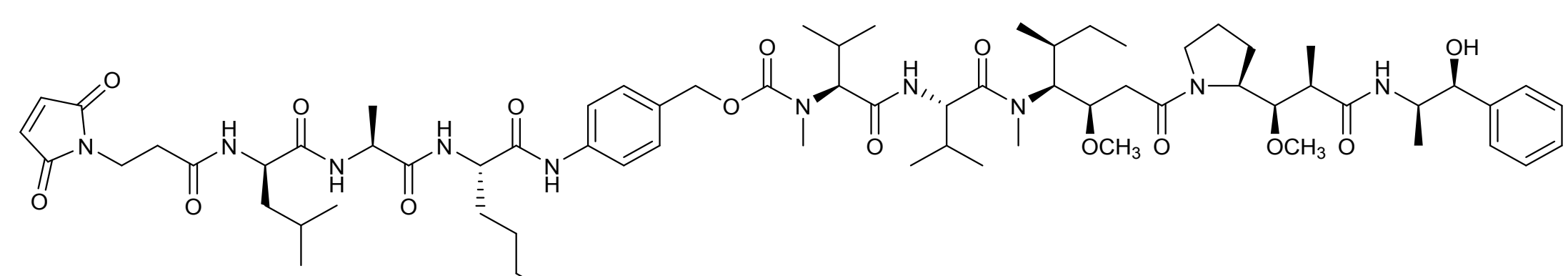

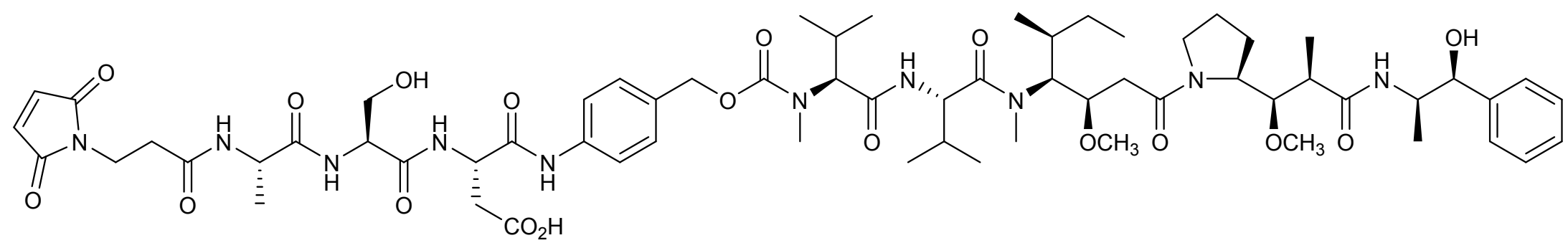

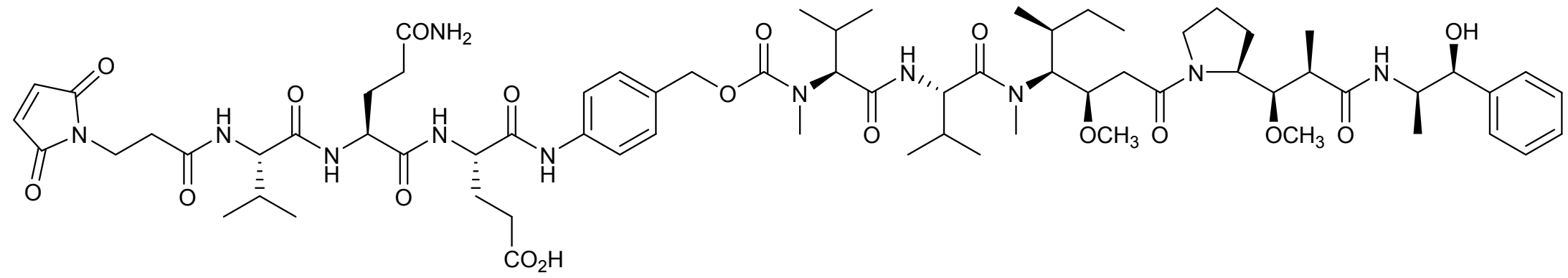

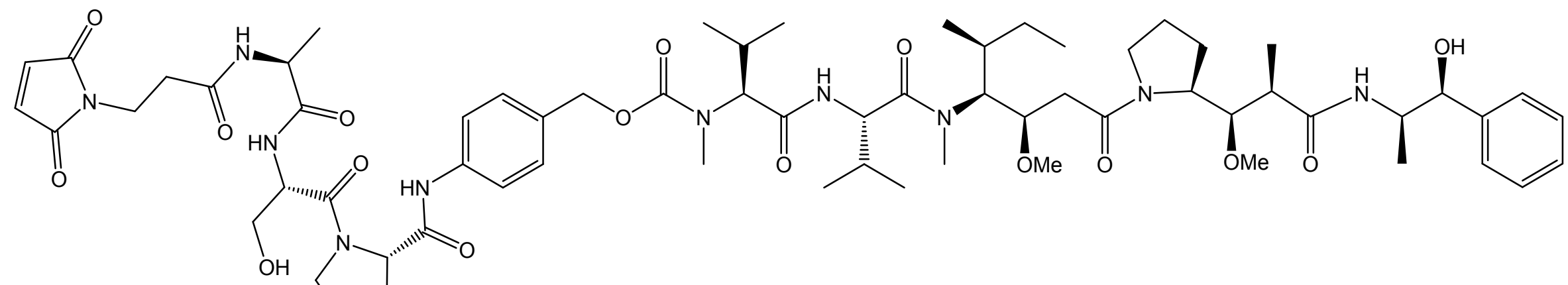

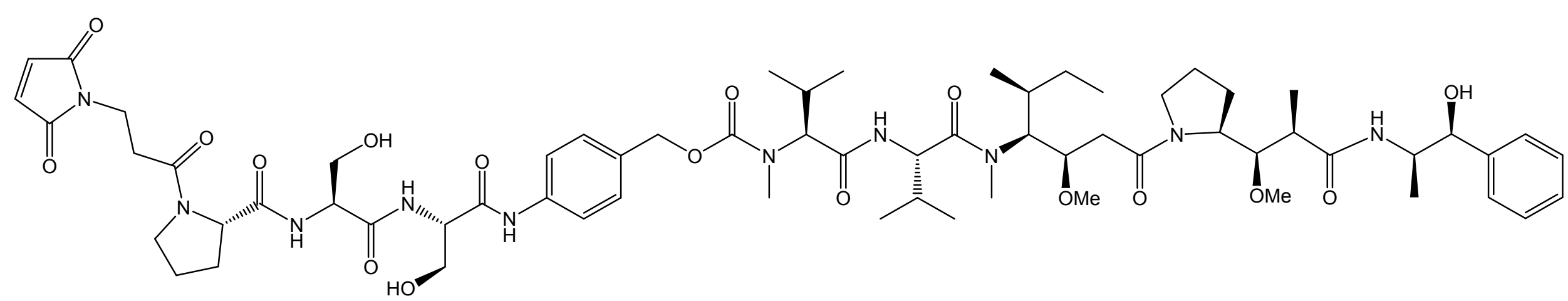

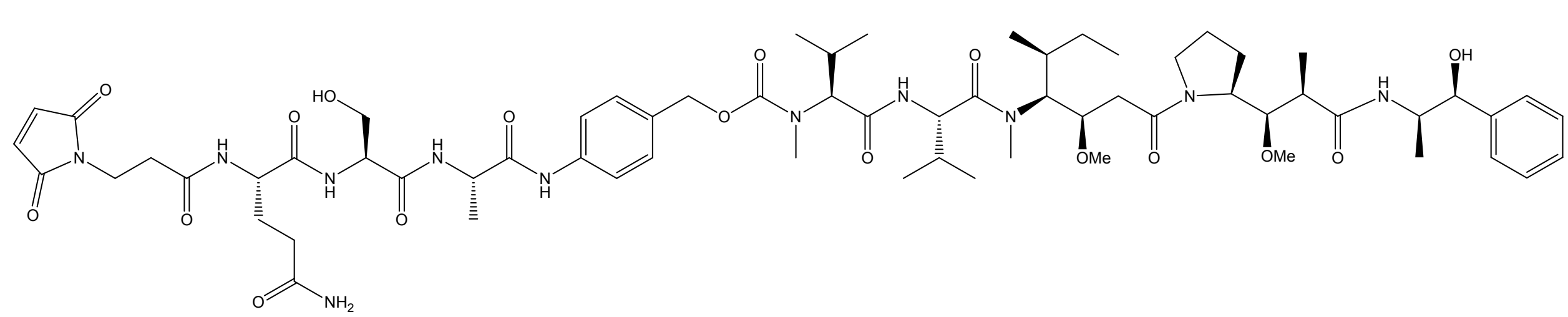

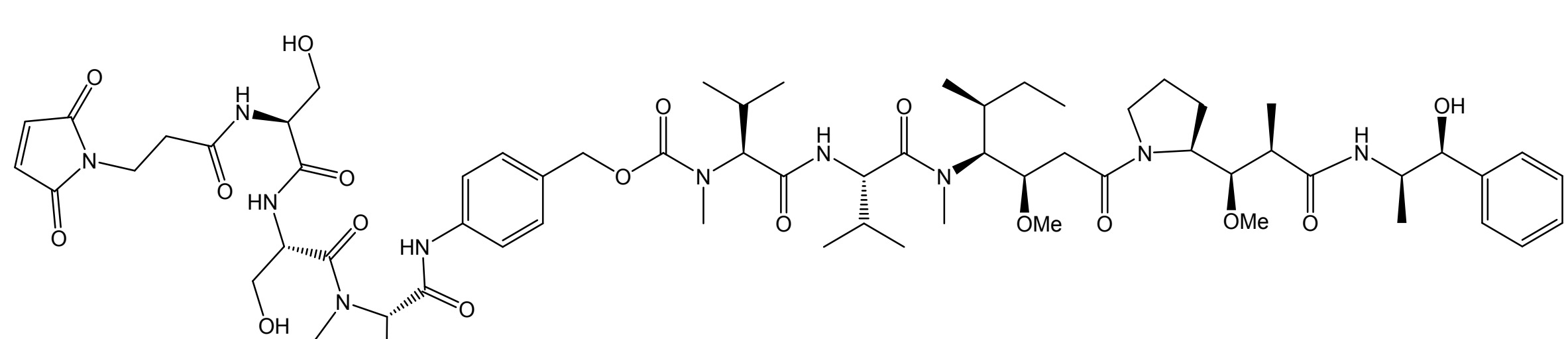

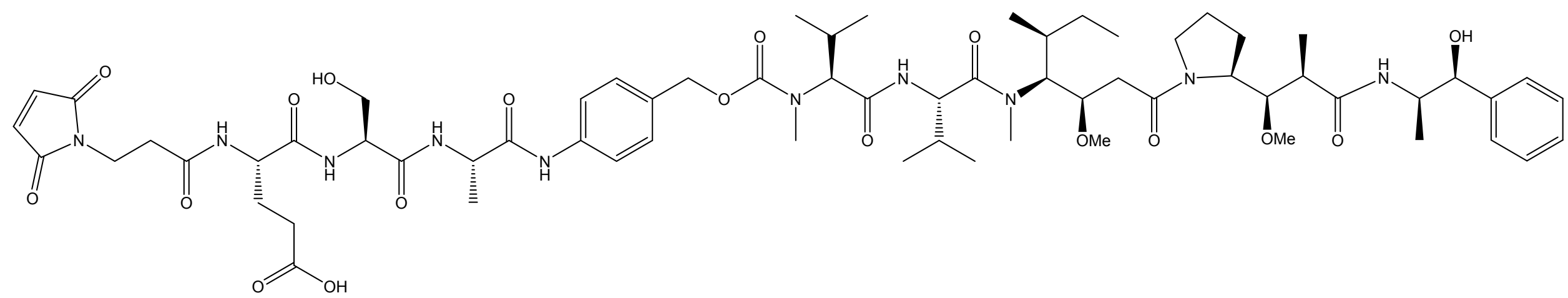

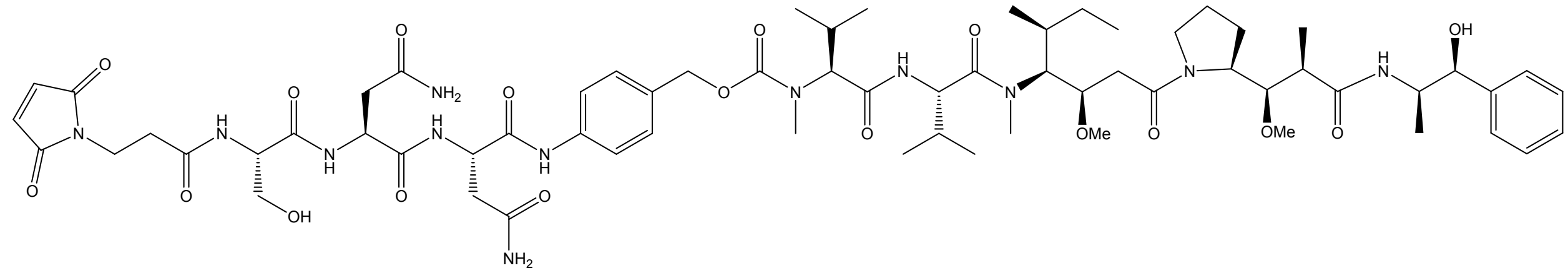

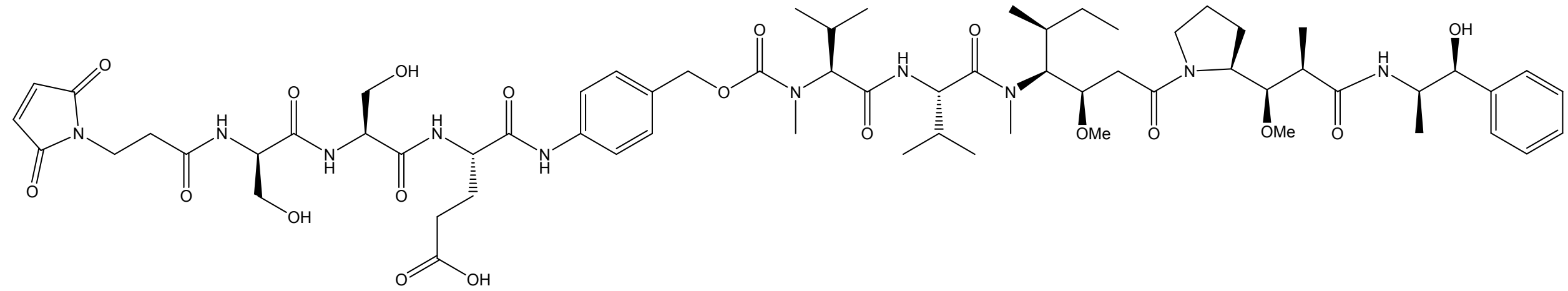

<u>Table 3</u>. Analytics of in vivo ADCs

Study ID	Antibody	Tripeptide* Sequence	DAR	DAR Analytic	% Monomer (SEC)
Rat ETS1	hIgG1	D-Leu-Leu-Met(O)	3.8	HIC	>95
Rat ETS1	hIgG1	D-Leu-Ala-Glu	3.8	HIC	>95
Rat ETS1	hIgG1	D-Leu-Leu-Cit	3.8	HIC	>95
Rat ETS1	hIgG1	Cit-Nal-Thr	3.9	HIC	>95
Rat ETS1	hIgG1	Pro-Nal-Lys	3.9	HIC	>95
Rat ETS1	hIgG1	D-Leu-Leu-Lys	3.8	HIC	>95
Rat ETS1	hIgG1	Vedotin	3.8	HIC	>95
Rat ETS1	hIgG1	mp-vcMMAE	4.0	HIC	>95
Rat ETS2	hIgG1	D-Leu-Ala-Glu	4.1	HIC	99.0
Rat ETS3	hIgG1	D-Leu-Ala-Cit	3.9	HIC	100
Rat-ETS3	hIgG1	Pro-Leu-Glu	3.9	HIC	99

Rat-ETS3	hIgG1	Thr-Glu-Leu	3.9	HIC	99
Rat-ETS3	hIgG1	D-Leu-Glu-Cit	4.0	HIC	99
Rat-ETS3	hIgG1	Glu-Pro-Cit	3.9	HIC	99
Rat-ETS3	hIgG1	O-allyl-Tyr-Pro-Cit	3.9	HIC	99
Rat-ETS3	hIgG1	Lys-Leu-Cit	3.9	HIC	98
Rat-ETS3	hIgG1	O-allyl-Tyr-DLeu-Glu	3.9	HIC	>98
Rat-ETS3	hIgG1	D-Leu-Ala-Gln	4.2	HIC	99
Rat-ETS3	hIgG1	D-Leu-Ala-(Se-Met)	4.0	HIC	99
Rat-ETS3	hIgG1	D-Leu-Glu-Ala	4.0	HIC	99
Rat-ETS3	hIgG1	D-Leu-Ala-Ala	4.0	HIC	99
Rat-ETS3	hIgG1	D-Leu-Ala-Met(O)	4.0	HIC	99
Rat-ETS3	hIgG1	D-Leu-Leu-Glu	4.0	HIC	99
Rat-ETS3	hIgG1	D-Ala-Ala-Glu	4.0	HIC	>98
Rat-ETS3	hIgG1	Ala-Ser-Glu	4.0	HIC	>98
Rat-ETS3	hIgG1	D-Leu-Ala-Asp	4.1	HIC	>98
Rat-ETS3	hIgG1	D-Leu-Val-Gln	3.9	HIC	>98
Rat-ETS3	hIgG1	D-Leu-Ala-Gla	4.1	HIC	>98
Rat-ETS3	hIgG1	D-Leu-Ala-Lys	4.1	HIC	>98
Rat-ETS3	hIgG1	Ala-Ser-Asp	4.2	HIC	>98
Rat-ETS3	hIgG1	Val-Gln-Glu	3.8	PLRP	99
Rat-ETS3	hIgG1	Ala-Ser-Pro	3.8	HIC	99
Rat-ETS3	hIgG1	Pro-Ser-Ser	3.8	HIC	98
Rat-ETS3	hIgG1	Gln-Ser-Ala	3.8	HIC	99
Rat-ETS3	hIgG1	Ser-Ser-Pro	3.8	PLRP	99
Rat-ETS3	hIgG1	Glu-Ser-Ala	3.8	HIC	99
Rat-ETS3	hIgG1	Ser-Asn-Asn	3.8	PLRP	98
Rat-ETS3	hIgG1	D-Ser-Ser-Glu	3.8	PLRP	98
Rat-ETS4	hIgG1	Leu-Ala-Glu	4.1	HIC	98
Rat-ETS4	hIgG1	D-Leu-Ala-Glu	4.1	HIC	99
Rat-ETS4	hIgG1	Vedotin	4.0	HIC	99
NHP-ETS1	hIgG1	Vedotin	4.1	PLRP	98
NHP-ETS1	hIgG1	D-Leu-Ala-Glu	4.2	PLRP	99
NHP-ETS2	Liv1-targeting	D-Leu-Ala-Glu	3.8	HIC	100

BxPC3	hIgG1	D-Leu-Ala-Glu	4.0	HIC	99%
BxPC3	Trop2-targeting	D-Leu-Ala-Glu	4.0	HIC	98%
BxPC3	Trop2-targeting	Vedotin	3.8	HIC	99%
MCF7(nci)	hIgG1	D-Leu-Ala-Glu	4.0	HIC	99%
MCF7(nci)	Trop2-targeting	D-Leu-Ala-Glu	4.0	HIC	98%
MCF7(nci)	Trop2-targeting	Vedotin	3.8	HIC	99%
MCF7(nci)	hIgG1	Vedotin	4.2	HIC	99%
MCF7(nci)	ALPP-targeting	Vedotin	4.1	HIC	100%
MCF7(nci)	ALPP-targeting	D-Leu-Ala-Glu	3.7	HIC	100%

*Abbreviations: Aib = α-aminoisobutyric acid, Cit = citrulline, Met(O) = methionine sulfoxide, Nal = naphthal-1-yl alanine, (Se-Met) = Selenomethionine, Gla = gamma-carboxyglutamate
